# Doubly Decarboxylative Synthesis of 4-(Pyridylmethyl)chroman-2-ones and 2-(Pyridylmethyl)chroman-4-ones under Mild Reaction Conditions †

**DOI:** 10.3390/molecules26154689

**Published:** 2021-08-03

**Authors:** Jan Bojanowski, Anna Albrecht

**Affiliations:** Institute of Organic Chemistry, Faculty of Chemistry, Lodz University of Technology, Żeromskiego 116, 90-924 Łódź, Poland; jan.bojanowski@dokt.p.lodz.pl

**Keywords:** decarboxylation, Michael addition, chromone-3-carboxylic acid, coumarin-3-carboxylic acid

## Abstract

The doubly decarboxylative Michael–type addition of pyridylacetic acid to chromone-3-carboxylic acids or coumarin-3-carboxylic acids has been developed. This protocol has been realized under Brønsted base catalysis, providing biologically interesting 4-(pyridylmethyl)chroman-2-ones and 2-(pyridylmethyl)chroman-4-ones in good or very good yields. The decarboxylative reaction pathway has been confirmed by mechanistic studies. Moreover, attempts to develop an enantioselective variant of the cascade are also described.

## 1. Introduction

Development of new C–C bond forming processes constitutes a fundamental task in the contemporary synthetic organic chemistry. The Michael reaction [1,2,3,4] is a very powerful means to accomplish this task, allowing access to various useful building blocks for organic synthesis [5,6,7,8,9,10,11]. Decarboxylative Michael reactions, where Michael acceptors and donors are activated through the presence of the carboxylic acid moiety, have recently emerged as a very useful strategy to access new reactivities [12,13,14,15,16,17,18,19,20,21,22,23,24,25,26,27,28,29,30]. In such a setup, carboxylate moiety serves a double purpose. It enhances the electrophilic or nucleophilic properties of the starting material and creates the opportunity for its facile removal via the decarboxylation process. One of the most common strategies, utilized to realize decarboxylative Michael reaction, was the activation of the Michael donors via decarboxylation with malonic acid half-thioesters (MAHT) and related systems being the most commonly employed [16,17,18,19,20,21,22,23].

**Scheme 1:**
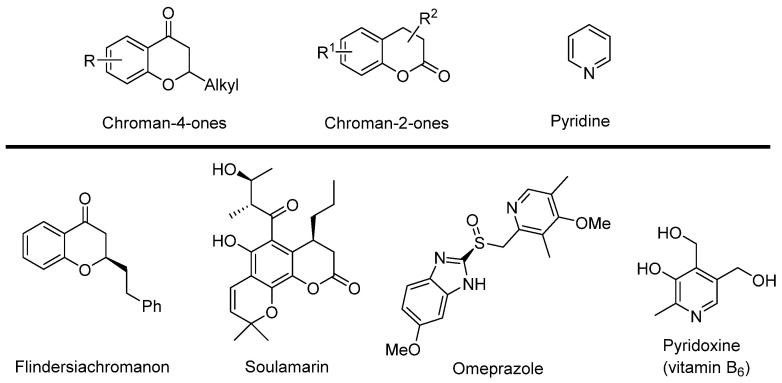
Representative examples of chroman-4-ones, chroman-2-ones and pyridine derivatives.

The pyridine and chromanone motifs are found in various natural products (Scheme 1). The chromanone ring system (chroman-2-on and chroman-4-on) and related compounds are found in different bioactive molecules relevant for the life-science industry [31,32,33,34,35,36,37,38,39,40]. Although these compounds are abundant in nature, synthetic methods for their preparation are not very common. On the other hand, pyridine is the second most frequent nitrogen-containing heterocyclic scaffold, and is present in 62 U.S. FDA approved drugs displaying a wide range of biological activities [41,42,43,44,45,46,47] with pyridine skeletons often serving as “privileged” scaffolds in drug design and discovery. Moreover, it is also a versatile building block utilized for the synthesis of chiral ligands applied in asymmetric catalysis [48,49,50]. In general, significant effort has been devoted to the synthesis of pyridine derivatives [49,50,51]. Representative examples of pyridine and chromanone derivatives are shown in Scheme 1.

**Scheme 2:**
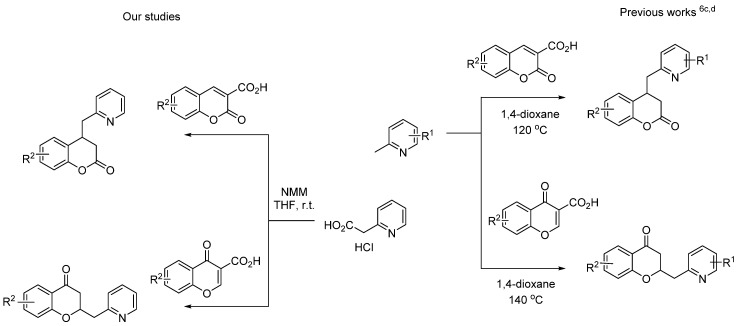
Previous studies and synthetic objective of the present work.

Given the importance of pyridine and chromanone chemistry for both academic and industrial purposes studies on the development of new doubly decarboxylative routes have been undertaken. It was anticipated that a new route to create hybrid molecules containing both structural fragments might rely on a doubly decarboxylative Michael reaction between 2-pyridylacetic acid **1** and coumarin-3-carboxylic acid **2** or chromone-3-carboxylic acids **4**. In this context it is worth mentioning that the addition of methyl pyridine to carboxylic acid group-activated coumarins and chromones was a subject of previous studies [6c,d]. However, very harsh reaction conditions (reactions were performed in 1,4-dioxane at high temperatures 120–140 °C) were required to accomplish this task (Scheme 2).

Herein, we present our studies on the reaction between coumarin-3-carboxylic acids **2** or chromone-3-carboxylic acids **3** (acting as electrophilic olefin) with 2-pyridylacetic acid hydrochloride **1** and related systems (acting as a pronucleophiles) proceeding under mild, basic conditions. Our studies demonstrate that activation of 2-methylpyridine through the presence of additional carboxylic-acid-group is beneficial for the process, providing an alternative method for the preparation of hybrid molecules containing pyridine and chromanone units.

## 2. Results

In optimization studies, coumarin-3-carboxylic acid **2a** or chromone-3-carboxylic acid **3a** and 2-pyridylacetic acid hydrochloride **1a** were selected as model reactants. The reaction was realized under basic conditions using 20 mol% excess of base over the hydrochloride). To our delight, in the presence of DABCO, the formation of target products **4a** or **5a** was observed (Table 1, entry 1). Adducts **4a** or **5a** were isolated in 69% and 23% yields, respectively. In the course of further studies, the influence of the base was checked (Table 1, entries 1–7). The yield of the reaction was improved to 77% (in the case of coumarin derivative **4a**) and 56% (in the case of 4-chromone derivatives **5a**) when *N*-methylmorpholine (NMM) was used (Table 1, entry 7). However, the formation of various products of substrate degradation was observed in the ^1^H NMR spectra of the crude reaction mixture samples, with the majority of side products being decarboxylated versions of the starting materials **1a**, **2a** and **3a**. Therefore, screening of the relative substrate ratio, thus taking into account their partial decomposition under reaction conditions, was undertaken (Table 1, entries 8–9). The use of 1,5-fold excess of 2-pyridylacetic acid hydrochloride **1** with respect to **2a** or **3a** proved the most effective yielding **4a** or **5a** in 98% or 70% yields, respectively (Table 1, entry 11). The amount of base was kept at 20 mol% while taking into account the amount needed to counterbalance the hydrochloride **1a** (Table 1, entry 11). In the next step, we turned our attention to solvent screening, but this did not improve the yields any further in both variants of the reaction (Table 1, entries 13–15). In the course of further studies, the use of coumarin **2b** or 4-chromone **3b** was evaluated (Table 1, entries 16 and 17). It was shown that starting materials **2b** or **3b** devoided of carboxylic groups moiety were inactive due to their decreased electrophilicity, which is consistent with the literature covering similar reactions [51]. Additionally, the control reaction of 2-methyl pyridine **1b** and **2a** in the presence of 0.2 equiv of *N*-methylmorpholine was performed (Table 1, entry 18) and no reaction was observed.

**Scheme 3:**
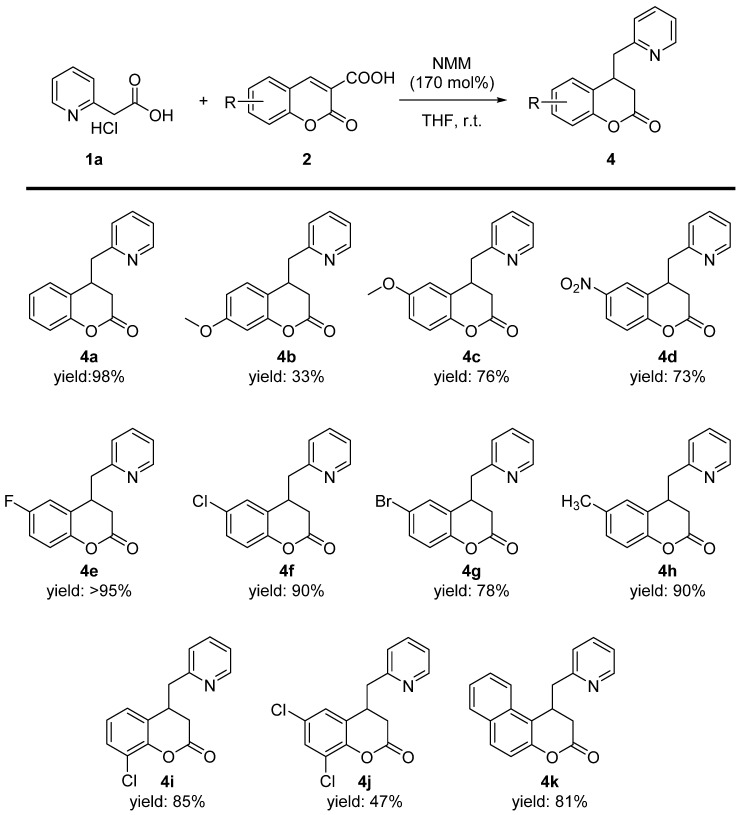
Decarboxylative synthesis of substituted 4-(2-pyridinylmethyl)coumarins **4**. Reactions performed on a 0.1 mmol scale using **2** (1.0 equiv.) and **1** (1.5 equiv.) in tetrahydrofuran (0.2 mL). For details, see Supplementary Materials.

**Scheme 4:**
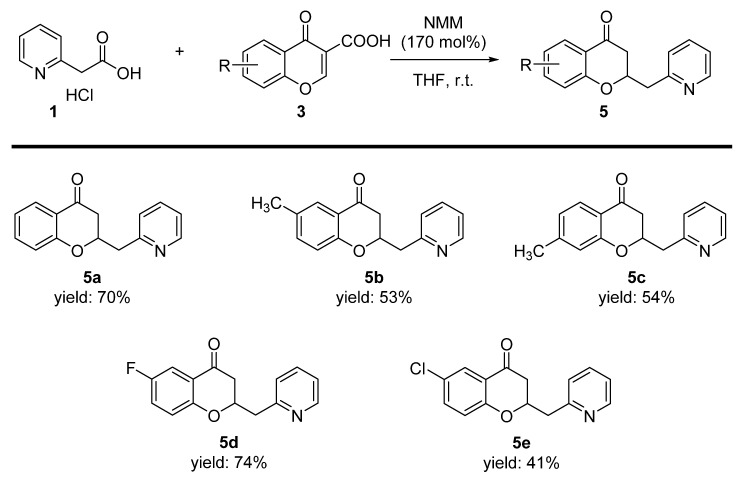
Decarboxylative synthesis of substituted 2-(4-pyridinylmethyl)chroman-4-ones **5**. Reactions performed on a 0.1 mmol scale using **3** (1.0 equiv.) and **1** (1.5 equiv.) in tetrahydrofuran (0.2 mL). For details see Supplementary Materials.

Having established the optimal reaction conditions, we turned our attention to the scope of the method by using various, substituted coumarin-3-carboxylic acids **2** or chromone-3-carboxylic acids **3** (Scheme 3 and Scheme 4). It was demonstrated that many substituents were well-tolerated in the case of the reaction with coumarins **2**, including electron-withdrawing, electron-donating groups, and bulky aromatic rings (Scheme 3). Only in the case of coumarin-3-carboxylic acid **2j** bearing two chloride atoms on the aromatic ring was lower yield observed.

In the course of further studies, the scope of the developed reactions with various chromone-3-carboxylic acids **3** was evaluated (Scheme 4). However, it was found that when electron-donating groups (acids **3b**, **3c**) as well as electron-withdrawing group (**3e**) were present in chromone-3-carboxylic acid moiety, the reaction proceeded with inferior results in comparison to their coumarin variant. 

**Scheme 5:**
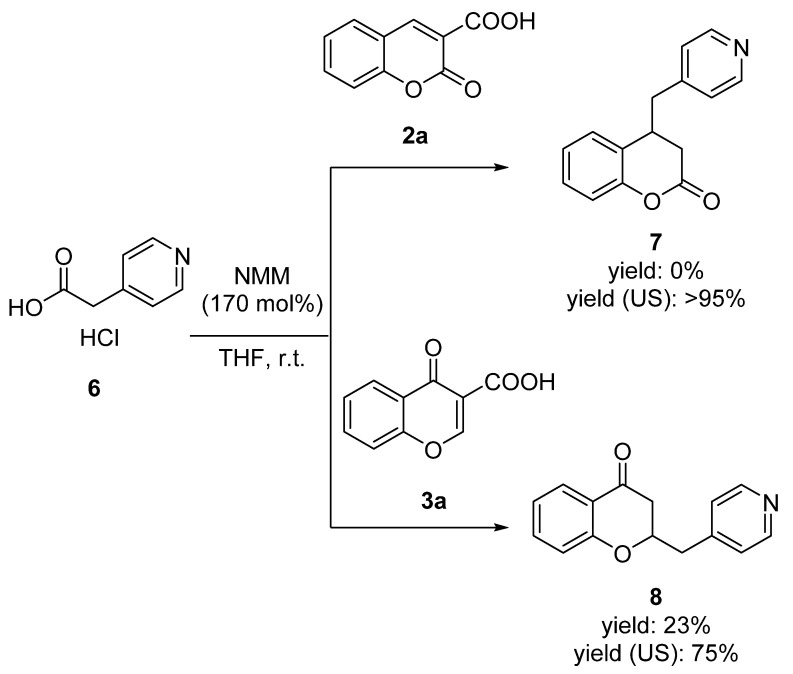
Decarboxylative synthesis of substituted (pyridyn-4-ylmethyl)chromanones **7**, **8**. Reactions were performed on a 0.1 mmol scale using **2a** or **3a** (1.0 equiv.) and **6** (1.5 equiv.) in 0.2 mL of tetrahydrofuran. US—ultrasound modification. For details see Supplementary Materials.

The scope of the method with regard to pronucleophiles turned out to be challenging, yielding no product when 4-pyridylacetic acid hydrochloride **6** was used in the reaction with coumarin-3-carboxylic acid **2** and only 23% yield of a corresponding chromone derivative **3**, mainly due to the formation of a gel which stopped the stirrer dipole and inhibited the reaction (Scheme 5). Interestingly, the ultrasound modification of the reaction helped to increase the yields substantially, achieving over 95% yield for **7** and 75% for **8**, respectively. In this modification the vial with a reaction mixture was subjected to ultrasound vibrations for 1.5 h. 

**Scheme 6:**
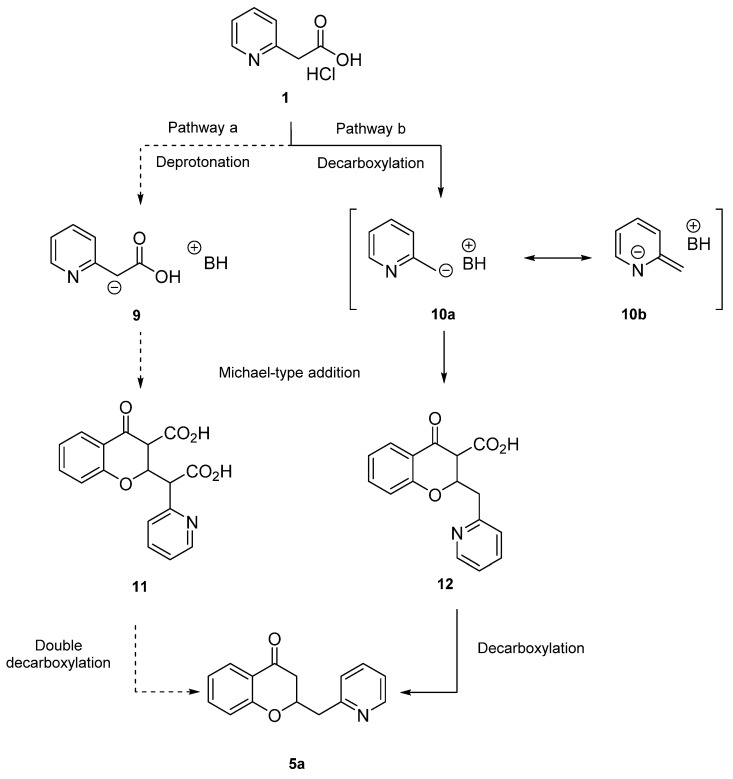
Mechanism of double decarboxylative synthesis of chroman-4-ones **5a**.

Notably, the developed process can proceed according to two mechanistic scenarios (Scheme 6). The first possibility concerns classical Michael-type addition of **9** generated from 2-pyridylacetic acid hydrochloride **1** to chromone-3-carboxylic acid **4a** followed by a double decarboxylation. On the other hand, the second possible mechanism involves two separate decarboxylation processes: (1) initial decarboxylation of 2-pyridylacetic acid hydrochloride **1** to give the corresponding carbanion **10** that undergoes Michael addition; (2) second decarboxylation of the Michael adduct to give target product **5a**. In order to confirm one of these two plausible reaction pathways, studies using mass spectrometry were performed. Interestingly, the molecular peak corresponding to the Michael adduct **12** was observed (cationic mode, calculated for C_16_H_13_NO_4_ [M + H]^+^: 284, found: 284). Furthermore, no molecular peak relating to **11** was detected in the spectra. Given these results, the mechanistic scenario involving decarboxylation, Michael addition and the second decarboxylation (Pathway b) is proposed as the one involved in the developed methodology.

**Scheme 7:**
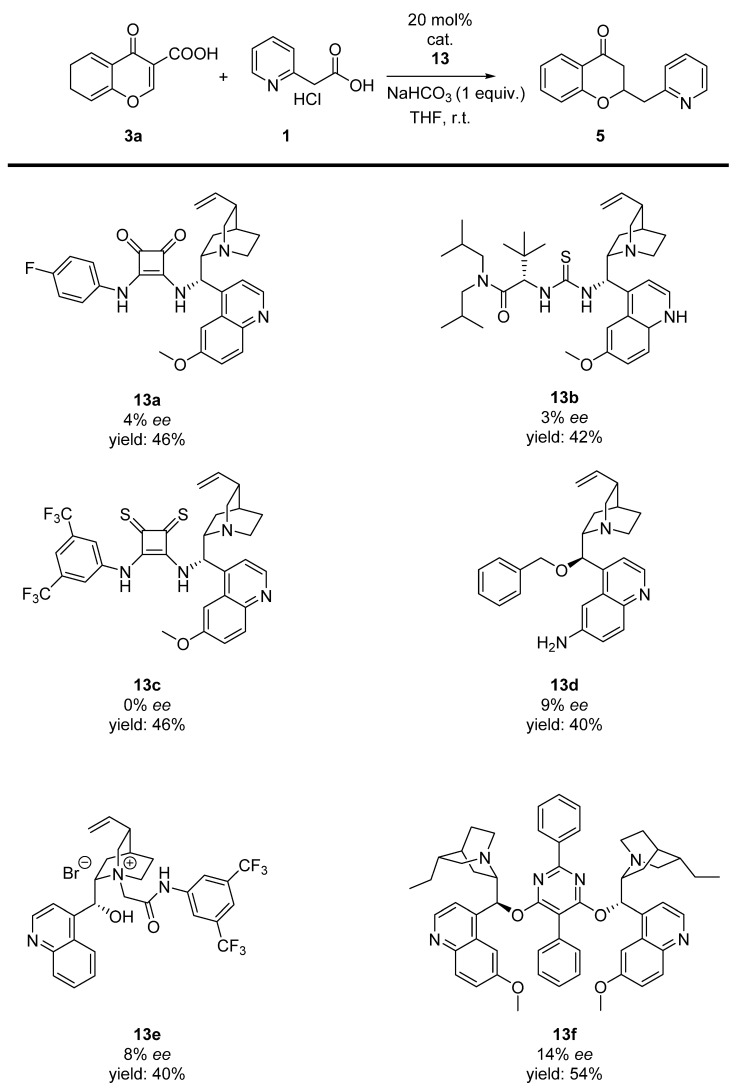
Chiral catalyst screening. The most successful catalyst in each structural category, respective enantiomeric excess and yield of the product obtained in the reaction.

Given the potential of the reaction, we turned our attention towards the development of enantioselective approach (Scheme 7). It was anticipated that by using cinchona alkaloids or their derivatives as Brønsted base catalysts in the studied reaction, access to enantiomerically enriched products should be possible. A model reaction of chromone-3-carboxylic acid **3a** and 2-pyridylacetic acid hydrochloride **1** was performed in the presence of different types of organocatalyst **13**: (**13a**) squaramides containing cinchona alkaloid derivatives; (**13b**) thioureas containing cinchona alkaloid derivatives; (**13c**) thiosquaramides containing cinchona alkaloid derivatives; (**13d**) protected and/or modified alkaloids without H-bond donor amides; (**13e**) phase-transfer catalysts; (**13f**) ethers containing two cinchona alkaloid moieties. Reaction was performed at room temperature and in tetrahydrofuran as a solvent. All tested catalysts promoted model reaction; however, enantioselectivity of the process was very low.

## 3. Conclusions

In conclusion, a new doubly decarboxylative Michael addition of pyridylacetic acid **1** to coumarin-3-carboxylic acids **2** or chromone-3-carboxylic acids **3** was developed. The scope studies confirmed the high efficiency of the transformation with regard to both coumarin-3-carboxylic acids and chromone-3-carboxylic acids providing access to a wide variety of interesting hybrid molecules bearing two important heterocyclic scaffolds: coumarin-pyridine and chromanone-pyridine ring systems. The decarboxylative reaction pathway has been confirmed by mechanistic studies. Initial studies on the development of asymmetric variants of the reaction have also been described.

## 4. Materials and Methods 

### 4.1. General Methods 

NMR spectra (See Supplementary Materials) were acquired on a Bruker Ultra Shield 700 instrument (Bruker Corporation, Billerica, MA, USA), running at 700 MHz for ^1^H and 176 MHz for ^13^C, respectively. Chemical shifts (δ) were reported in ppm relative to residual solvent signals (CDCl_3_: 7.26 ppm for ^1^H NMR, 77.16 ppm for ^13^C NMR). Mass spectra were recorded on a Bruker Maxis Impact spectrometer using electrospray (ES+) ionization (referenced to the mass of the charged species). Analytical thin layer chromatography (TLC) was performed using pre-coated aluminium-backed plates (Merck Kieselgel 60 F254) and visualized by ultraviolet irradiation. Unless otherwise noted, analytical grade solvents and commercially available reagents were used without further purification. For flash chromatography (FC), silica gel (Silica gel 60, 230–400 mesh, Merck, Darmstadt, Germany) was used. The enantiomeric ratio (er) of the products was determined by chiral stationary phase HPLC (Daicel Chiralpak IA column). 2-Pyridylacetic acid hydrochloride **1** and 4-Pyridylacetic acid hydrochloride **6** were used as commercially-available reagents. Chromone-3-carboxylic acids **2** and coumarin-3-carboxylic acids **3** were prepared from the corresponding 2-hydroxyacetophenones following the literature procedure [52,53]. 

### 4.2. General Procedure 

An ordinary screw-cap vial was charged with a magnetic stirring bar; the corresponding coumarin-3-carboxylic acid **2** or chromone-3-carboxylic acid **3** (0.1 mmol, 1 equivalent), THF (0.2 mL), *N*-methyl morpholine (0.17 mmol, 1.7 equivalent), and pyridylacetic acid hydrochloride **1** or **6** (0.15 mmol, 1.5 equivalent) was added. The reaction mixture was stirred at room temperature and monitored by ^1^H NMR spectroscopy. After the complete consumption of the carboxylic acid **2a** or **3a**, the mixture was directly subjected to FC on silica gel (*n*-hexane:ethyl acetate 3:1 or 2:1) to afford the pure products **4**, **5** or **7**. 

The ultrasound variant of the reaction proceeded under the same reaction conditions, however without a stirring bar and with the shorter reaction time of 1.5 h.

*4-[(Pyridin-2-yl)methyl]-3,4-dihydro-2H-1-benzopyran-2-one* (**4a**) pure product was isolated by flash chromatography on silica gel (*n*-hexane:ethyl acetate—3:1) as colorless oil in over 98% yield following the regular procedure and 59% yield following the ultrasound procedure. ^1^H NMR (700 MHz, Chloroform-d) δ 8.60 (ddd, *J* = 4.8, 1.8, 0.9 Hz, 1H), 7.57 (td, *J* = 7.6, 1.8 Hz, 1H), 7.27–7.23 (m, 1H), 7.17 (ddd, *J* = 7.5, 4.9, 1.1 Hz, 1H), 7.07 (d, *J* = 8.0 Hz, 1H), 7.06–7.02 (m, 2H), 6.96 (d, *J* = 7.7 Hz, 1H), 3.68 (dddd, *J* = 8.8, 6.9, 5.9, 3.7 Hz, 1H), 3.11 (dd, *J* = 13.8, 6.9 Hz, 1H), 2.94 (dd, *J* = 13.8, 8.8 Hz, 1H), 2.79 (dd, *J* = 16.0, 5.9 Hz, 1H), 2.73 (dd, *J* = 16.0, 3.7 Hz, 1H). ^13^C NMR (176 MHz, Chloroform-d) δ 168.1, 157.9, 151.5, 149.8, 136.7, 128.6, 127.8, 126.2, 124.5, 124.3, 122.0, 117.2, 43.2, 35.4, 34.2. 

*7-Methoxy-4-[(pyridin-2-yl)methyl]-3,4-dihydro-2H-1-benzopyran-2-one* (**4b**) pure product was isolated by flash chromatography on silica gel (*n*-hexane:ethyl acetate—3:1) as colorless oil in 33% yield. ^1^H NMR (700 MHz, Chloroform-*d*) δ 8.59 (ddd, *J* = 4.9, 1.8, 0.9 Hz, 1H), 7.57 (td, *J* = 7.6, 1.8 Hz, 1H), 7.16 (ddd, *J* = 7.5, 4.9, 1.1 Hz, 1H), 6.95 (dt, *J* = 7.7, 1.0 Hz, 1H), 6.90 (d, *J* = 8.5 Hz, 1H), 6.62 (d, *J* = 2.5 Hz, 1H), 6.58 (dd, *J* = 8.4, 2.6 Hz, 1H), 3.78 (s, 3H), 3.60 (dddd, *J* = 8.6, 7.0, 6.0, 3.8 Hz, 1H), 3.07 (dd, *J* = 13.7, 7.0 Hz, 1H), 2.91 (dd, *J* = 13.7, 8.6 Hz, 1H), 2.77 (dd, *J* = 15.9, 6.0 Hz, 1H), 2.71 (dd, *J* = 15.9, 3.8 Hz, 1H). ^13^C NMR (176 MHz, Chloroform-*d*) δ 168.2, 159.9, 158.1, 152.3, 149.8, 136.7, 128.4, 124.4, 122.0, 118.0, 110.6, 102.8, 55.7, 43.6, 34.8, 34.5.

*6-Methoxy-4-[(pyridin-2-yl)methyl]-3,4-dihydro-2H-1-benzopyran-2-one* (**4c**) pure product was isolated by flash chromatography on silica gel (*n*-hexane:ethyl acetate—3:1) as colorless oil in 76% yield following the regular procedure and 69% yield following the ultrasound procedure. ^1^H NMR (700 MHz, Chloroform-*d*) δ 8.60 (ddd, *J* = 4.9, 1.8, 0.9 Hz, 1H), 7.59 (td, *J* = 7.6, 1.8 Hz, 1H), 7.18 (ddd, *J* = 7.5, 4.9, 1.1 Hz, 1H), 7.00 (d, *J* = 8.9 Hz, 1H), 6.98 (dt, *J* = 7.8, 1.0 Hz, 1H), 6.78 (dd, *J* = 8.9, 3.0 Hz, 1H), 6.55 (d, *J* = 3.0 Hz, 1H), 3.70 (s, 3H), 3.63 (dddd, *J* = 8.8, 6.9, 5.9, 3.6 Hz, 1H), 3.10 (dd, *J* = 13.7, 6.9 Hz, 1H), 2.93 (dd, *J* = 13.7, 8.8 Hz, 1H), 2.76 (dd, *J* = 16.0, 5.9 Hz, 1H), 2.70 (dd, *J* = 16.0, 3.6 Hz, 1H). ^13^C NMR (176 MHz, Chloroform-*d*) δ 168.3, 158.0, 156.2, 149.8, 145.4, 136.7, 127.0, 124.4, 122.0, 118.0, 114.1, 112.5, 55.7, 43.1, 35.7, 34.1.

*6-Nitro-4-[(pyridin-2-yl)methyl]-3,4-dihydro-2H-1-benzopyran-2-one* (**4d**) pure product was isolated by flash chromatography on silica gel (*n*-hexane:ethyl acetate—2:1) as colorless oil in 73% yield. ^1^H NMR (700 MHz, Chloroform-*d*) δ 8.60 (d, *J* = 4.2 Hz, 1H), 8.14 (dd, *J* = 8.9, 2.7 Hz, 1H), 7.97 (d, *J* = 2.6 Hz, 1H), 7.60 (td, *J* = 7.6, 1.8 Hz, 1H), 7.21 (dd, *J* = 7.1, 5.2 Hz, 1H), 7.18 (d, *J* = 8.9 Hz, 1H), 6.99 (d, *J* = 7.7 Hz, 1H), 3.84 (dddd, *J* = 8.3, 7.1, 5.6, 4.3 Hz, 1H), 3.14 (dd, *J* = 14.0, 7.0 Hz, 1H), 3.01 (dd, *J* = 14.0, 8.3 Hz, 1H), 2.88–2.81 (m, 2H). ^13^C NMR (176 MHz, Chloroform-*d*) δ 166.2, 156.8, 155.9, 149.8, 144.2, 137.1, 127.2, 124.6, 124.2, 124.0, 122.5, 118.1, 42.6, 35.1, 33.6.

*6-Fluoro-4-[(pyridin-2-yl)methyl]-3,4-dihydro-2H-1-benzopyran-2-one* (**4e**) pure product was isolated by flash chromatography on silica gel (hexane:ethyl acetate—3:1) as colorless oil in over 95% yield. ^1^H NMR (700 MHz, Chloroform-*d*) δ 8.59 (ddd, *J* = 4.9, 1.8, 0.9 Hz, 1H), 7.58 (td, *J* = 7.6, 1.8 Hz, 1H), 7.18 (ddd, *J* = 7.5, 4.9, 1.1 Hz, 1H), 7.02 (dd, *J* = 8.9, 4.6 Hz, 1H), 6.97 (d, *J* = 7.7 Hz, 1H), 6.93 (ddd, *J* = 8.8, 8.0, 3.0 Hz, 1H), 6.73 (dd, *J* = 8.4, 3.0 Hz, 1H), 3.67 (dddd, *J* = 8.6, 7.0, 5.8, 4.0 Hz, 1H), 3.09 (dd, *J* = 13.8, 7.0 Hz, 1H), 2.93 (dd, *J* = 13.9, 8.6 Hz, 1H), 2.76 (dd, *J* = 16.1, 5.8 Hz, 1H), 2.72 (dd, *J* = 16.1, 4.0 Hz, 1H). ^13^C NMR (176 MHz, Chloroform-*d*) δ 167.7, 159.1 (d, *J* = 244.1 Hz), 157.5, 149.8, 147.5 (d, *J* = 2.5 Hz), 136.8, 127.9 (d, *J* = 7.8 Hz), 124.3, 122.2, 118.5 (d, *J* = 8.4 Hz), 115.3 (d, *J* = 23.6 Hz), 114.4 (d, *J* = 24.0 Hz), 42.8, 35.3, 33.9.

*6-Chloro-4-[(pyridin-2-yl)methyl]-3,4-dihydro-2H-1-benzopyran-2-one* (**4f**) pure product was isolated by flash chromatography on silica gel (*n*-hexane:ethyl acetate—2:1) as colorless oil in 90% yield following the regular procedure and 79% yield following the ultrasound procedure. ^1^H NMR (700 MHz, Chloroform-*d*) δ 8.60 (ddd, *J* = 4.9, 1.8, 0.9 Hz, 1H), 7.60 (td, *J* = 7.6, 1.8 Hz, 1H), 7.22 (dd, *J* = 8.6, 2.5 Hz, 1H), 7.19 (ddd, *J* = 7.5, 4.8, 1.1 Hz, 1H), 7.03 (d, *J* = 2.5 Hz, 1H), 7.01 (d, *J* = 8.6 Hz, 1H), 6.97 (dt, *J* = 7.7, 1.0 Hz, 1H), 3.67 (dddd, *J* = 8.7, 6.8, 5.8, 4.0 Hz, 1H), 3.10 (dd, *J* = 13.9, 6.8 Hz, 1H), 2.93 (dd, *J* = 13.9, 8.7 Hz, 1H), 2.77 (dd, *J* = 16.1, 5.8 Hz, 1H), 2.73 (dd, *J* = 16.1, 4.0 Hz, 1H). ^13^C NMR (176 MHz, Chloroform-*d*) δ 167.4, 157.5, 150.1, 149.8, 136.8, 129.6, 128.7, 127.9, 127.8, 124.3, 122.2, 118.6, 42.9, 35.3, 33.8.

*6-Bromo-4-[(pyridin-2-yl)methyl]-3,4-dihydro-2H-1-benzopyran-2-one* (**4g**) pure product was isolated by flash chromatography on silica gel (*n*-hexane:ethyl acetate—3:1) as colorless oil in 78% yield. ^1^H NMR (700 MHz, Chloroform-*d*) δ 8.59 (ddd, *J* = 4.9, 1.8, 0.9 Hz, 1H), 7.59 (td, *J* = 7.6, 1.8 Hz, 1H), 7.36 (dd, *J* = 8.6, 2.4 Hz, 1H), 7.18 (ddd, *J* = 7.6, 4.9, 1.1 Hz, 1H), 7.17 (d, *J* = 2.3 Hz, 1H), 6.97 (d, *J* = 7.7 Hz, 1H), 6.94 (d, *J* = 8.6 Hz, 1H), 3.66 (dddd, *J* = 8.8, 6.8, 5.8, 4.0 Hz, 1H), 3.09 (dd, *J* = 13.8, 6.8 Hz, 1H), 2.92 (dd, *J* = 13.9, 8.8 Hz, 1H), 2.76 (dd, *J* = 16.1, 5.8 Hz, 1H), 2.72 (dd, *J* = 16.1, 4.0 Hz, 1H). 13C NMR (176 MHz, Chloroform-d) δ 167.4, 157.4, 150.6, 149.9, 136.8, 131.6, 130.7, 128.3, 124.3, 122.2, 119.0, 117.1, 42.9, 35.2, 33.8.

*6-Methyl-4-[(pyridin-2-yl)methyl]-3,4-dihydro-2H-1-benzopyran-2-one* (**4h**) pure product was isolated by flash chromatography on silica gel (*n*-hexane:ethyl acetate—3:1) as colorless oil in 90% yield. ^1^H NMR (700 MHz, Chloroform-*d*) δ 8.60 (d, *J* = 4.5 Hz, 1H), 7.58 (td, *J* = 7.6, 1.7 Hz, 1H), 7.17 (dd, *J* = 7.3, 5.0 Hz, 1H), 7.04 (dd, *J* = 7.2, 1.5 Hz 1H), 6.97 (d, *J* = 7.7 Hz, 1H), 6.95 (d, *J* = 8.2 Hz, 1H), 6.85 (s, 1H), 3.60 (dddd, *J* = 9.1, 6.1, 5.9, 3.7 Hz, 1H), 3.10 (dd, *J* = 13.7, 6.5 Hz, 1H), 2.89 (dd, *J* = 13.7, 9.1 Hz, 1H), 2.74 (dd, *J* = 16.0, 5.9 Hz, 1H), 2.69 (dd, *J* = 16.0, 3.7 Hz, 1H), 2.25 (s, 3H). ^13^C NMR (176 MHz, Chloroform-*d*) δ 168.4, 158.1, 149.8, 149.4, 136.6, 134.1, 129.1, 128.2, 125.9, 124.4, 122.0, 116.9, 43.3, 35.4, 34.1, 20.8.

*8-Chloro-4-[(pyridin-2-yl)methyl]-3,4-dihydro-2H-1-benzopyran-2-one* (**4i**) pure product was isolated by flash chromatography on silica gel (*n*-hexane:ethyl acetate—2:1) as colorless oil in 85% yield. ^1^H NMR (700 MHz, Chloroform-*d*) δ 8.60 (ddd, *J* = 4.8, 1.8, 0.9 Hz, 1H), 7.58 (td, *J* = 7.6, 1.8 Hz, 1H), 7.32 (dd, *J* = 7.9, 1.6 Hz, 1H), 7.18 (ddd, *J* = 7.5, 4.9, 1.1 Hz, 1H), 6.98–6.95 (m, 2H), 6.92 (ddd, *J* = 7.6, 1.6, 0.6 Hz, 1H), 3.72 (dddd, *J* = 8.7, 7.0, 5.7, 3.7 Hz, 1H), 3.09 (dd, *J* = 13.8, 7.0 Hz, 1H), 2.95 (dd, *J* = 13.8, 8.7 Hz, 1H), 2.80 (dd, *J* = 16.0, 5.7 Hz, 1H), 2.76 (dd, *J* = 16.0, 3.7 Hz, 1H). ^13^C NMR (176 MHz, Chloroform-*d*) δ 166.8, 157.6, 149.8, 147.4, 136.8, 129.4, 128.1, 126.2, 124.8, 124.4, 122.2, 122.1, 43.0, 35.7, 33.9.

*6,8-Dichloro-4-[(pyridin-2-yl)methyl]-3,4-dihydro-2H-1-benzopyran-2-one* (**4j**) pure product was isolated by flash chromatography on silica gel (*n*-hexane:ethyl acetate—2:1) as colorless oil in 47% yield. ^1^H NMR (700 MHz, Chloroform-*d*) δ 8.60 (ddd, *J* = 4.8, 1.7, 0.9 Hz, 1H), 7.61 (td, *J* = 7.6, 1.8 Hz, 1H), 7.33 (d, *J* = 2.4 Hz, 1H), 7.20 (ddd, *J* = 7.5, 4.9, 1.0 Hz, 1H), 6.98 (d, *J* = 7.7 Hz, 1H), 6.93 (dd, *J* = 2.4, 0.6 Hz, 1H), 3.71 (dddd, *J* = 8.7, 6.9, 5.3, 4.1 Hz, 1H), 3.08 (dd, *J* = 13.9, 6.9 Hz, 1H), 2.94 (dd, *J* = 13.9, 8.7 Hz, 1H), 2.81–2.74 (m, 1H). ^13^C NMR (176 MHz, Chloroform-*d*) δ 166.1, 157.1, 149.9, 146.3, 136.9, 129.5, 129.2, 129.2, 126.3, 124.3, 123.1, 122.3, 42.7, 35.6, 33.6.

*1-[(Pyridin-2-yl)methyl]-1H,2H,3H-naphtho[2,1-b]pyran-3-one* (**4k**) pure product was isolated by flash chromatography on silica gel (*n*-hexane:ethyl acetate—3:1) as colorless oil in 81% yield. ^1^H NMR (700 MHz, Chloroform-*d*) δ 8.65 (ddd, *J* = 4.8, 1.8, 0.9 Hz, 1H), 8.00 (d, *J* = 8.4 Hz, 1H), 7.84 (d, *J* = 8.1 Hz, 1H), 7.79 (d, *J* = 8.8 Hz, 1H), 7.55–7.50 (m, 2H), 7.45 (ddd, *J* = 8.0, 6.8, 1.1 Hz, 1H), 7.26 (d, *J* = 8.8 Hz, 1H), 7.17 (ddd, *J* = 7.5, 4.9, 0.9 Hz, 1H), 6.97 (d, *J* = 7.7 Hz, 1H), 4.33 (dddd, *J* = 10.1, 6.3, 5.1, 1.8 Hz, 1H), 3.24 (dd, *J* = 14.0, 5.1 Hz, 1H), 2.92 (dd, *J* = 14.1, 10.1 Hz, 1H), 2.88 (dd, *J* = 16.1, 1.8 Hz, 1H), 2.81 (ddd, *J* = 16.1, 6.3, 0.8 Hz, 1H). ^13^C NMR (176 MHz, Chloroform-*d*) δ 168.1, 158.0, 149.9, 149.2, 136.7, 131.1, 130.7, 129.4, 128.8, 127.4, 125.3, 124.5, 122.9, 122.0, 119.6, 117.6, 42.1, 33.3, 31.9.

*2-[(Pyridin-2-yl)methyl]-3,4-dihydro-2H-1-benzopyran-4-one* (**5a**) pure product was isolated by flash chromatography on silica gel (*n*-hexane:ethyl acetate—3:1) as yellow oil in 70% yield following the regular procedure and in 65% yield following the ultrasound procedure. ^1^H NMR (700 MHz, Chloroform-*d*) δ 8.56 (ddd, *J* = 4.9, 1.8, 0.9 Hz, 1H), 7.88 (ddd, *J* = 7.8, 1.8, 0.4 Hz, 1H), 7.65 (td, *J* = 7.7, 1.8 Hz, 1H), 7.45 (ddd, *J* = 8.4, 7.2, 1.8 Hz, 1H), 7.28–7.25 (m, 1H), 7.19 (ddd, *J* = 7.5, 4.9, 1.1 Hz, 1H), 7.00 (ddd, *J* = 7.9, 7.2, 1.1 Hz, 1H), 6.93 (ddd, *J* = 8.4, 1.0, 0.4 Hz, 1H), 4.96 (dddd, *J* = 9.7, 7.0, 6.0, 5.7 Hz, 1H), 3.39 (dd, *J* = 13.9, 7.0 Hz, 1H), 3.20 (dd, *J* = 13.9, 6.0 Hz, 1H), 2.79–2.73 (m, 2H). ^13^C NMR (176 MHz, Chloroform-*d*) δ 192.2, 161.5, 156.8, 149.7, 136.6, 136.1, 127.1, 124.4, 122.1, 121.5, 121.2, 118.1, 77.5, 43.5, 42.7.

*6-Methyl-2-[(pyridin-2-yl)methyl]-3,4-dihydro-2H-1-benzopyran-4-one* (**5b**) pure product was isolated by flash chromatography on silica gel (*n*-hexane:ethyl acetate—3:1) as yellow oil in 53% yield. ^1^H NMR (700 MHz, Chloroform-*d*) δ 8.56 (dd, *J* = 4.8, 1.7 Hz, 1H), 7.68–7.62 (m, 2H), 7.27–7.25 (m, 2H), 7.18 (ddd, *J* = 7.6, 4.9, 1.2 Hz, 1H), 6.83 (d, *J* = 8.5 Hz, 1H), 4.91 (ddt, *J* = 9.2, 7.0, 6.1 Hz, 1H), 3.37 (dd, *J* = 13.9, 7.0 Hz, 1H), 3.18 (dd, *J* = 13.9, 6.0 Hz, 1H), 2.76–2.70 (m, 2H), 2.29 (s, 3H). ^13^C NMR (176 MHz, Chloroform-*d*) δ 192.5, 159.6, 157.0, 149.7, 137.2, 136.6, 130.9, 126.7, 124.4, 122.0, 120.8, 117.9, 77.4, 43.6, 42.7, 20.5.

*7-Methyl-2-[(pyridin-2-yl)methyl]-3,4-dihydro-2H-1-benzopyran-4-one* (**5c**) pure product was isolated by flash chromatography on silica gel (*n*-hexane:ethyl acetate—3:1) as yellow oil in 54% yield. ^1^H NMR (700 MHz, Chloroform-*d*) δ 8.56 (ddd, *J* = 4.9, 1.8, 0.9 Hz, 1H), 7.76 (d, *J* = 8.0 Hz, 1H), 7.65 (td, *J* = 7.7, 1.8 Hz, 1H), 7.26 (d, *J* = 7.8 Hz, 1H), 7.18 (ddd, *J* = 7.5, 4.9, 1.1 Hz, 1H), 6.81 (ddd, *J* = 8.0, 1.5, 0.6 Hz, 1H), 6.73 (bs, 1H), 4.92 (dtd, *J* = 8.1, 7.1, 6.0 Hz, 1H), 3.36 (dd, *J* = 13.9, 7.1 Hz, 1H), 3.18 (dd, *J* = 13.9, 5.9 Hz, 1H), 2.75–2.67 (m, 2H), 2.33 (s, 3H). ^13^C NMR (176 MHz, Chloroform-*d*) δ 191.9, 161.6, 157.0, 149.7, 147.6, 136.6, 127.0, 124.4, 122.9, 122.0, 119.0, 118.1, 77.5, 43.6, 42.6, 22.0.

*6-Fluoro-2-[(pyridin-2-yl)methyl]-3,4-dihydro-2H-1-benzopyran-4-one* (**5d**) pure product was isolated by flash chromatography on silica gel (*n*-hexane:ethyl acetate—3:1) as colorless oil in 74%. ^1^H NMR (700 MHz, Chloroform-*d*) δ 8.55 (ddd, *J* = 4.9, 1.7, 0.9 Hz, 1H), 7.66 (td, *J* = 7.7, 1.8 Hz, 1H), 7.51 (dd, *J* = 8.2, 3.2 Hz, 1H), 7.25 (d, *J* = 7.0 Hz, 1H), 7.19 (ddd, *J* = 7.5, 4.9, 1.1 Hz, 1H), 7.16 (ddd, *J* = 9.0, 7.7, 3.2 Hz, 1H), 6.90 (dd, *J* = 9.1, 4.2 Hz, 1H), 4.93 (dddd, *J* = 11.6, 6.9, 6.0, 3.9 Hz, 1H), 3.37 (dd, *J* = 14.0, 6.9 Hz, 1H), 3.19 (dd, *J* = 14.0, 6.0 Hz, 1H), 2.77 (dd, *J* = 16.9, 3.9 Hz, 1H), 2.73 (dd, *J* = 16.9, 11.7 Hz, 1H). ^13^C NMR (176 MHz, Chloroform-*d*) δ 191.4 (d, *J* = 1.6 Hz), 157.8 (d, *J* = 1.6 Hz), 157.3 (d, *J* = 241.1 Hz), 156.6, 149.7, 136.7, 124.3, 123.6 (d, *J* = 24.8 Hz), 122.1, 121.6 (d, *J* = 6.5 Hz), 119.7 (d, *J* = 9.5 Hz), 112.0 (d, *J* = 23.5 Hz), 77.7, 43.4, 42.4.

*6-Chloro-2-[(pyridin-2-yl)methyl]-3,4-dihydro-2H-1-benzopyran-4-one* (**5e**) pure product was isolated by flash chromatography on silica gel (*n*-hexane:ethyl acetate—2:1) as yellow oil in 41% yield following the regular procedure. The reaction did not yield the desired product following the ultrasound procedure. ^1^H NMR (700 MHz, Chloroform-*d*) δ 8.56 (d, *J* = 5.3 Hz, 1H), 7.82 (d, *J* = 2.6 Hz, 1H), 7.66 (td, *J* = 7.7, 1.8 Hz, 1H), 7.38 (dd, *J* = 8.8, 2.7 Hz, 1H), 7.25 (d, *J* = 7.0 Hz, 1H), 7.20 (dd, *J* = 7.8, 4.6 Hz, 1H), 6.89 (d, *J* = 8.8 Hz, 1H), 4.95 (dddd, *J* = 11.8, 6.9, 6.0, 3.8 Hz, 1H), 3.37 (dd, *J* = 14.0, 6.9 Hz, 1H), 3.20 (dd, *J* = 14.0, 6.1 Hz, 1H), 2.78 (dd, *J* = 16.9, 3.9 Hz, 1H), 2.74 (dd, *J* = 16.9, 11.8 Hz, 1H). ^13^C NMR (176 MHz, Chloroform-*d*) δ 191.0, 160.0, 156.6, 149.8, 136.7, 135.9, 127.1, 126.5, 124.3, 122.2, 122.0, 119.9, 77.8, 43.4, 42.4.

*4-[(Pyridin-4-yl)methyl]-3,4-dihydro-2H-1-benzopyran-2-one* (**7**) pure product was isolated by flash chromatography on silica gel (*n*-hexane:ethyl acetate—3:1) as colorless oil in over 95% yield following the ultrasound procedure. The regular procedure did not yield the desired product. ^1^H NMR (700 MHz, Chloroform-*d*) δ 8.52–8.51 (m, 2H), 7.28 (ddd, *J* = 8.1, 7.6, 1.6 Hz, 1H), 7.08 (dd, *J* = 8.1, 1.1 Hz, 1H), 7.04 (td, *J* = 7.5, 1.2 Hz, 1H), 7.01–6.98 (m, 2H), 6.93 (dd, *J* = 7.5, 1.6 Hz, 1H), 3.26 (dddd, *J* = 8.5, 7.4, 5.8, 3.5 Hz, 1H), 2.91 (dd, *J* = 13.6, 7.4 Hz, 1H), 2.81–2.75 (m, 2H), 2.73 (dd, *J* = 16.0, 3.5 Hz, 1H). ^13^C NMR (176 MHz, Chloroform-*d*) δ 167.5, 151.4, 150.2, 146.9, 129.0, 127.9, 125.1, 124.6, 124.6, 117.4, 40.7, 36.8, 34.2.

*2-[(Pyridin-4-yl)methyl]-3,4-dihydro-2H-1-benzopyran-4-one* (**8**) pure product was isolated by flash chromatography on silica gel (*n*-hexane:ethyl acetate—3:1) as yellow oil in 23% yield following the regular procedure and in 75% yield following the ultrasound procedure. ^1^H NMR (700 MHz, Chloroform-*d*) δ 8.58–8.52 (m, 2H), 7.86 (ddd, *J* = 7.8, 1.8, 0.4 Hz, 1H), 7.47 (ddd, *J* = 8.4, 7.2, 1.8 Hz, 1H), 7.24–7.18 (m, 2H), 7.02 (ddd, *J* = 7.9, 7.2, 1.1 Hz, 1H), 6.94 (ddd, *J* = 8.4, 1.0, 0.4 Hz, 1H), 4.71 (dddd, *J* = 9.6, 7.3, 5.9, 5.2 Hz, 1H), 3.16 (dd, *J* = 14.2, 7.4 Hz, 1H), 3.04 (dd, *J* = 14.2, 5.1 Hz, 1H), 2.74–2.67 (m, 2H). ^13^C NMR (176 MHz, Chloroform-*d*) δ 191.6, 161.2, 150.2 (2C), 145.5, 136.3, 127.2, 125.0 (2C), 121.8, 121.1, 118.1, 77.3, 42.6, 40.6.

## Figures and Tables

**Table 1:** Optimization studies of doubly decarboxylative synthesis of 4-(2-pyridylmethyl)chroman-2-ones **4** and 2-(4-pyridylmethyl)chroman-4-ones **5** ^a^.

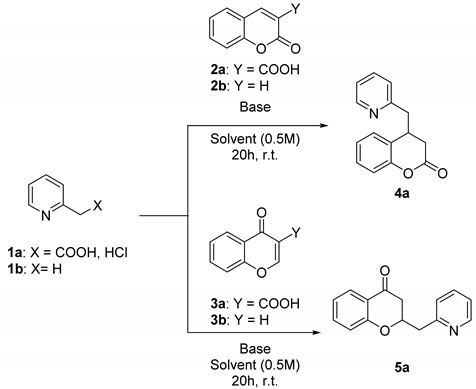								
No.	Solvent	Base (equiv.)	X	Y	2 or 3 (equiv)	1a (equiv.)	Yield of 4a	Yield of 5a
1	THF	DABCO (1.2)	COOH	COOH	1.0	1.0	69	23
2	THF	DMAP (1.2)	COOH	COOH	1.0	1.0	62	50
3	THF	Pyrrolidine (1.2)	COOH	COOH	1.0	1.0	65	50
4	THF	2,6-lutidine (1.2)	COOH	COOH	1.0	1.0	54	38
5	THF	TEA (1.2)	COOH	COOH	1.0	1.0	71	21
6	THF	DIPEA (1.2)	COOH	COOH	1.0	1.0	74	42
7	THF	NMM (1.2)	COOH	COOH	1.0	1.0	77	56
8	THF	NMM (1.2)	COOH	COOH	1.5	1.0	71	63
9	THF	NMM (1.2)	COOH	COOH	1.2	1.0	47	52
10	THF	NMM (1.4)	COOH	COOH	1.0	1.2	82	61
11	THF	NMM (1.7)	COOH	COOH	1.0	1.5	**98**	**70**
12	THF	NMM (2.2)	COOH	COOH	1.0	2.0	92	70
13	Toluene	NMM (1.7)	COOH	COOH	1.0	1.5	19	40
14	MeCN	NMM (1.7)	COOH	COOH	1.0	1.5	75	69
15	CH_2_Cl_2_	NMM (1.7)	COOH	COOH	1.0	1.5	75	54
16	THF	NMM (1.7)	COOH	H	**2b** (1.0)	1.5	no reaction	-
17	THF	NMM (1.7)	COOH	H	**3b** (1.0)	1.5	-	no reaction
18	THF	NMM (0.2)	H	COOH	**2a** (1.0)	**1b** (1.5)	no reaction	-

^a^ Reactions were performed on a 0.1 mmol scale in 0.2 mL of the solvent.

## Data Availability

Data are contained within the article.

## Supplementary Materials

The following are available online.

## Sample Availability

Samples of the compounds are available from the authors.
