# Supplementary material for: Doubly Decarboxylative Synthesis of 4-(Pyridylmethyl)chroman-2-ones and 2-(Pyridylmethyl)chroman-4-ones under Mild Reaction Conditions
Source: Molecules. 2021 Aug 3;26(15):4689. doi: 10.3390/molecules26154689 (PMC8347554; doi:10.3390/molecules26154689)

Doubly decarboxylative synthesis of 4-(pyridylmethyl)chroman-2-ones and 2-(pyridylmethyl)chroman-4-ones under mild conditions.

Jan Bojanowski [1], Anna Albrecht [1,*]

[1] Institute of Organic Chemistry, Faculty of Chemistry, Lodz University of Technology, Żeromskiego 116, 90-924 Łódź, Poland
* Correspondence: anna.albrecht@p.lodz.pl

Table of contents

1. General Methods 1

2. General Procedure - Reactions with carboxylic acids 2

3. Enantioselective synthesis of 2-[(pyridin-2-yl)methyl]-3,4-dihydro-2H-1-benzopyran-4-one **5a**……………………………………………………………………………………………..6

4. Mechanistic considerations using MS analysis 8

5. NMR spectra of products 10

1. General Methods

NMR spectra were acquired on a Bruker Ultra Shield 700 instrument (Bruker Corporation, Billerica, MA, USA), running at 700 MHz for ^{1}H and 176 MHz for ^{13}C, respectively. Chemical shifts (δ) were reported in ppm relative to residual solvent signals ($CDCl_3$: 7.26 ppm for ^{1}H NMR, 77.16 ppm for ^{13}C NMR). Mass spectra were recorded on a Bruker Maxis Impact spectrometer using electrospray (ES+) ionization (referenced to the mass of the charged species). Analytical thin layer chromatography (TLC) was performed using pre-coated aluminium-backed plates (Merck Kieselgel 60 F254) and visualized by the ultraviolet irradiation. Unless otherwise noted, analytical grade solvents and commercially available reagents were used without further purification. For flash chromatography (FC), silica gel (Silica gel 60, 230–400 mesh, Merck, Darmstadt, Germany) was used. The enantiomeric ratio (er) of the products were determined by chiral stationary phase HPLC (Daicel Chiralpak IA column). Pyridylacetic acid hydrochlorides **1** and **6** were used as commercially available reagents. Coumarin-3-carboxylic acids 2 and chromone-3-carboxylic acids **4** were prepared following the literature procedure [1,2].

1. Ishizuka, N.; Matsunori, K.; Sakai, K.; Fujimoto, M.; Mihara, S.; Yamamori, T. Structure−Activity Relationships of a Novel Class of Endothelin-A Receptor Antagonists and Discovery of Potent and Selective Receptor Antagonist, 2-(Benzo[1,3]dioxol-5-yl)-6-isopropyloxy-4-(4-methoxyphenyl)-2H-chromene-3- carboxylic Acid (S-1255). 1. Study on Structure−Activity Relationships and Basic Structure Crucial for ETA Antagonism. *J. Med. Chem.* **2002**, *45*, 2041–2055 DOI: 10.1021/jm010382z

2. Song, A.; Wang, X. ; Lam, K. S. A convenient synthesis of coumarin-3-carboxylic acids via Knoevenagel condensation of Meldrum's acid with ortho-hydroxyaryl aldehydes or ketones. *Tetrahedron Lett.* **2003**, 44, 1755-1758. DOI: 10.1016/S0040-4039(03)00108-4

2. General Procedure - Reactions with carboxylic acids

An ordinary screw-cap vial was charged with a magnetic stirring bar, the corresponding coumarin-3-carboxylic acids **2** or chromone-3-carboxylic acids **3** (0.1 mmol, 1 equivalent), THF (0.2 mL), *N*-methyl morpholine (0.17 mmol, 1.7 equivalent), and the corresponding pyridylacetic acid hydrochloride **1** or **6** (0.15 mmol, 1.5 equivalent). The reaction mixture was stirred at room temperature and monitored by ^{1}H NMR spectroscopy. After the complete consumption of the carboxylic acid **2** or **3**, the mixture was directly subjected to FC on silica gel (*n*-hexane:ethyl acetate 3:1 or 2:1) to afford pure products **4** or **5**.

Ultrasound variant of the reaction proceeded under the same conditions, however without the need of using a stirring bar and with the reaction time shortened to 1.5 hours.

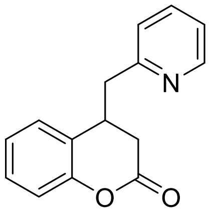

4-[(Pyridin-2-yl)methyl]-3,4-dihydro-2*H*-1-benzopyran-2-one (4a) pure product was isolated by flash chromatography on silica gel (*n*-hexane : ethyl acetate – 3 : 1) as colorless oil in over 98% yield following regular procedure and 59% yield following ultrasound procedure. ^{1}H NMR (700 MHz, Chloroform-d) δ 8.60 (ddd, *J* = 4.8, 1.8, 0.9 Hz, 1H), 7.57 (td, *J* = 7.6, 1.8 Hz, 1H), 7.27 – 7.23 (m, 1H), 7.17 (ddd, *J* = 7.5, 4.9, 1.1 Hz, 1H), 7.07 (d, *J* = 8.0 Hz, 1H), 7.06 – 7.02 (m, 2H), 6.96 (d, *J* = 7.7 Hz, 1H), 3.68 (dddd, *J* = 8.8, 6.9, 5.9, 3.7 Hz, 1H), 3.11 (dd, *J* = 13.8, 6.9 Hz, 1H), 2.94 (dd, *J* = 13.8, 8.8 Hz, 1H), 2.79 (dd, *J* = 16.0, 5.9 Hz, 1H), 2.73 (dd, *J* = 16.0, 3.7 Hz, 1H). 13C NMR (176 MHz, Chloroform-d) δ 168.1, 157.9, 151.5, 149.8, 136.7, 128.6, 127.8, 126.2, 124.5, 124.3, 122.0, 117.2, 43.2, 35.4, 34.2. HRMS: Calculated for [$C_{15}H_{13}NO_2$+H^+]: 240.1019, found 240.1024.

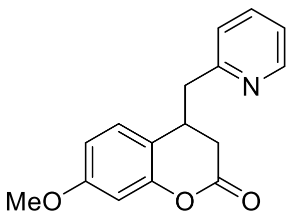

7-Methoxy-4-[(pyridin-2-yl)methyl]-3,4-dihydro-2*H*-1-benzopyran-2-one (4b) pure product was isolated by flash chromatography on silica gel (*n*-hexane : ethyl acetate – 3 : 1) as colorless oil in 33% yield. ^{1}H NMR (700 MHz, Chloroform-*d*) δ 8.59 (ddd, *J* = 4.9, 1.8, 0.9 Hz, 1H), 7.57 (td, *J* = 7.6, 1.8 Hz, 1H), 7.16 (ddd, *J* = 7.5, 4.9, 1.1 Hz, 1H), 6.95 (dt, *J* = 7.7, 1.0 Hz, 1H), 6.90 (d, *J* = 8.5 Hz, 1H), 6.62 (d, *J* = 2.5 Hz, 1H), 6.58 (dd, *J* = 8.4, 2.6 Hz, 1H), 3.78 (s, 3H), 3.60 (dddd, *J* = 8.6, 7.0, 6.0, 3.8 Hz, 1H), 3.07 (dd, *J* = 13.7, 7.0 Hz, 1H), 2.91 (dd, *J* = 13.7, 8.6 Hz, 1H), 2.77 (dd, *J* = 15.9, 6.0 Hz, 1H), 2.71 (dd, *J* = 15.9, 3.8 Hz, 1H). ^{13}C NMR (176 MHz, Chloroform-*d*) δ 168.2, 159.9, 158.1, 152.3, 149.8, 136.7, 128.4, 124.4, 122.0, 118.0, 110.6, 102.8, 55.7, 43.6, 34.8, 34.5. HRMS: Calculated for [$C_{16}H_{15}NO_3$+H^+]: 270.1125, found 270.1128.

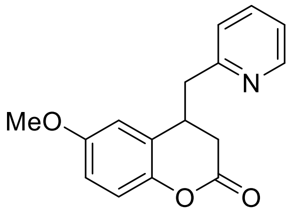

6-Methoxy-4-[(pyridin-2-yl)methyl]-3,4-dihydro-2*H*-1-benzopyran-2-one (4c) pure product was isolated by flash chromatography on silica gel (*n*-hexane : ethyl acetate – 3 : 1) as colorless oil in 76% yield following regular procedure and 69% yield following ultrasound procedure. ^{1}H NMR (700 MHz, Chloroform-*d*) δ 8.60 (ddd, *J* = 4.9, 1.8, 0.9 Hz, 1H), 7.59 (td, *J* = 7.6, 1.8 Hz, 1H), 7.18 (ddd, *J* = 7.5, 4.9, 1.1 Hz, 1H), 7.00 (d, *J* = 8.9 Hz, 1H), 6.98 (dt, *J* = 7.8, 1.0 Hz, 1H), 6.78 (dd, *J* = 8.9, 3.0 Hz, 1H), 6.55 (d, *J* = 3.0 Hz, 1H), 3.70 (s, 3H), 3.63 (dddd, *J* = 8.8, 6.9, 5.9, 3.6 Hz, 1H), 3.10 (dd, *J* = 13.7, 6.9 Hz, 1H), 2.93 (dd, *J* = 13.7, 8.8 Hz, 1H), 2.76 (dd, *J* = 16.0, 5.9 Hz, 1H), 2.70 (dd, *J* = 16.0, 3.6 Hz, 1H). ^{13}C NMR (176 MHz, Chloroform-*d*) δ 168.3, 158.0, 156.2, 149.8, 145.4, 136.7, 127.0, 124.4, 122.0, 118.0, 114.1, 112.5, 55.7, 43.1, 35.7, 34.1. HRMS: Calculated for [$C_{16}H_{15}NO_3$+H^+]: 270.1125, found 270.1128.

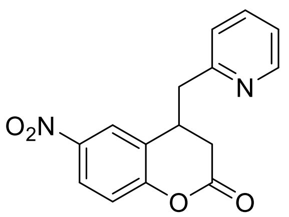

6-Nitro-4-[(pyridin-2-yl)methyl]-3,4-dihydro-2*H*-1-benzopyran-2-one (4d) pure product was isolated by flash chromatography on silica gel (*n*-hexane : ethyl acetate – 2 : 1) as colorless oil in 73% yield. ^{1}H NMR (700 MHz, Chloroform-*d*) δ 8.60 (d, *J* = 4.2 Hz, 1H), 8.14 (dd, *J* = 8.9, 2.7 Hz, 1H), 7.97 (d, *J* = 2.6 Hz, 1H), 7.60 (td, *J* = 7.6, 1.8 Hz, 1H), 7.21 (dd, *J* = 7.1, 5.2 Hz, 1H), 7.18 (d, *J* = 8.9 Hz, 1H), 6.99 (d, *J* = 7.7 Hz, 1H), 3.84 (dddd, *J* = 8.3, 7.1, 5.6, 4.3 Hz, 1H), 3.14 (dd, *J* = 14.0, 7.0 Hz, 1H), 3.01 (dd, *J* = 14.0, 8.3 Hz, 1H), 2.88 – 2.81 (m, 2H). ^{13}C NMR (176 MHz, Chloroform-*d*) δ 166.2, 156.8, 155.9, 149.8, 144.2, 137.1, 127.2, 124.6, 124.2, 124.0, 122.5, 118.1, 42.6, 35.1, 33.6. HRMS: Calculated for [$C_{15}H_{12}N_2O_4$+H^+]: 285.0870, found 285.0875.

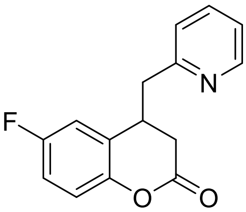

6-Fluoro-4-[(pyridin-2-yl)methyl]-3,4-dihydro-2*H*-1-benzopyran-2-one (4e) pure product was isolated by flash chromatography on silica gel (*n*-hexane : ethyl acetate – 3 : 1) as colorless oil in over 95% yield. ^1H NMR (700 MHz, Chloroform-*d*) δ 8.59 (ddd, *J* = 4.9, 1.8, 0.9 Hz, 1H), 7.58 (td, *J* = 7.6, 1.8 Hz, 1H), 7.18 (ddd, *J* = 7.5, 4.9, 1.1 Hz, 1H), 7.02 (dd, *J* = 8.9, 4.6 Hz, 1H), 6.97 (d, *J* = 7.7 Hz, 1H), 6.93 (ddd, *J* = 8.8, 8.0, 3.0 Hz, 1H), 6.73 (dd, *J* = 8.4, 3.0 Hz, 1H), 3.67 (dddd, *J* = 8.6, 7.0, 5.8, 4.0 Hz, 1H), 3.09 (dd, *J* = 13.8, 7.0 Hz, 1H), 2.93 (dd, *J* = 13.9, 8.6 Hz, 1H), 2.76 (dd, *J* = 16.1, 5.8 Hz, 1H), 2.72 (dd, *J* = 16.1, 4.0 Hz, 1H). ^{13}C NMR (176 MHz, Chloroform-*d*) δ 167.7, 159.1 (d, *J* = 244.1 Hz), 157.5, 149.8, 147.5 (d, *J* = 2.5 Hz), 136.8, 127.9 (d, *J* = 7.8 Hz), 124.3, 122.2, 118.5 (d, *J* = 8.4 Hz), 115.3 (d, *J* = 23.6 Hz), 114.4 (d, *J* = 24.0 Hz), 42.8, 35.3, 33.9. HRMS: Calculated for [$C_{15}H_{12}FNO_2+H^+$]: 258.0925, found 258.0931.

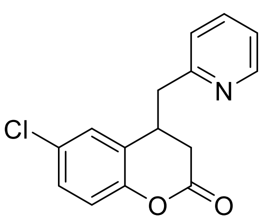

6-Chloro-4-[(pyridin-2-yl)methyl]-3,4-dihydro-2*H*-1-benzopyran-2-one (4f) pure product was isolated by flash chromatography on silica gel (*n*-hexane : ethyl acetate – 2 : 1) as colorless oil in 90% yield following regular procedure and 79% yield following ultrasound procedure. ^1H NMR (700 MHz, Chloroform-*d*) δ 8.60 (ddd, *J* = 4.9, 1.8, 0.9 Hz, 1H), 7.60 (td, *J* = 7.6, 1.8 Hz, 1H), 7.22 (dd, *J* = 8.6, 2.5 Hz, 1H), 7.19 (ddd, *J* = 7.5, 4.8, 1.1 Hz, 1H), 7.03 (d, *J* = 2.5 Hz, 1H), 7.01 (d, *J* = 8.6 Hz, 1H), 6.97 (dt, *J* = 7.7, 1.0 Hz, 1H), 3.67 (dddd, *J* = 8.7, 6.8, 5.8, 4.0 Hz, 1H), 3.10 (dd, *J* = 13.9, 6.8 Hz, 1H), 2.93 (dd, *J* = 13.9, 8.7 Hz, 1H), 2.77 (dd, *J* = 16.1, 5.8 Hz, 1H), 2.73 (dd, *J* = 16.1, 4.0 Hz, 1H). ^{13}C NMR (176 MHz, Chloroform-*d*) δ 167.4, 157.5, 150.1, 149.8, 136.8, 129.6, 128.7, 127.9, 127.8, 124.3, 122.2, 118.6, 42.9, 35.3, 33.8. HRMS: Calculated for [$C_{15}H_{12}ClNO_2+H^+$]: 274.0629, found 274.0637.

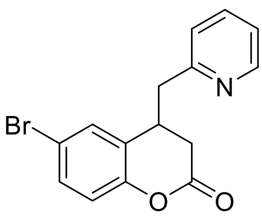

6-Bromo-4-[(pyridin-2-yl)methyl]-3,4-dihydro-2*H*-1-benzopyran-2-one (4g) pure product was isolated by flash chromatography on silica gel (*n*-hexane : ethyl acetate – 3 : 1) as colorless oil in 78% yield. ^1H NMR (700 MHz, Chloroform-*d*) δ 8.59 (ddd, *J* = 4.9, 1.8, 0.9 Hz, 1H), 7.59 (td, *J* = 7.6, 1.8 Hz, 1H), 7.36 (dd, *J* = 8.6, 2.4 Hz, 1H), 7.18 (ddd, *J* = 7.6, 4.9, 1.1 Hz, 1H), 7.17 (d, J = 2.3 Hz, 1H), 6.97 (d, J = 7.7 Hz, 1H), 6.94 (d, J = 8.6 Hz, 1H), 3.66 (dddd, J = 8.8, 6.8, 5.8, 4.0 Hz, 1H), 3.09 (dd, J = 13.8, 6.8 Hz, 1H), 2.92 (dd, J = 13.9, 8.8 Hz, 1H), 2.76 (dd, J = 16.1, 5.8 Hz, 1H), 2.72 (dd, J = 16.1, 4.0 Hz, 1H). 13C NMR (176 MHz, Chloroform-d) δ 167.4, 157.4, 150.6, 149.9, 136.8, 131.6, 130.7, 128.3, 124.3, 122.2, 119.0, 117.1, 42.9, 35.2, 33.8. HRMS: Calculated for [$C_{15}H_{12}BrNO_2+H^+$]: 318.0124, found 318.0136.

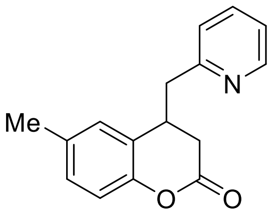

6-Methyl-4-[(pyridin-2-yl)methyl]-3,4-dihydro-2*H*-1-benzopyran-2-one (4h) pure product was isolated by flash chromatography on silica gel (*n*-hexane : ethyl acetate – 3 : 1) as colorless oil in 90% yield. ^1H NMR (700 MHz, Chloroform-*d*) δ 8.60 (d, *J* = 4.5 Hz, 1H), 7.58 (td, *J* = 7.6, 1.7 Hz, 1H), 7.17 (dd, *J* = 7.3, 5.0 Hz, 1H), 7.04 (dd, *J* = 7.2, 1.5 Hz 1H), 6.97 (d, *J* = 7.7 Hz, 1H), 6.95 (d, *J* = 8.2 Hz, 1H), 6.85 (s, 1H), 3.60 (dddd, *J* = 9.1, 6.1, 5.9, 3.7 Hz, 1H), 3.10 (dd, *J* = 13.7, 6.5 Hz, 1H), 2.89 (dd, *J* = 13.7, 9.1 Hz, 1H), 2.74 (dd, *J* = 16.0, 5.9 Hz, 1H), 2.69 (dd, *J* = 16.0, 3.7 Hz, 1H), 2.25 (s, 3H). ^{13}C NMR (176 MHz, Chloroform-*d*) δ 168.4, 158.1, 149.8, 149.4, 136.6, 134.1, 129.1, 128.2, 125.9, 124.4, 122.0, 116.9, 43.3, 35.4, 34.1, 20.8. HRMS: Calculated for [$C_{16}H_{15}NO_2+H^+$]: 254.1176, found 254.1183.

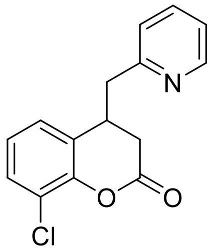

8-Chloro-4-[(pyridin-2-yl)methyl]-3,4-dihydro-2*H*-1-benzopyran-2-one (4i) pure product was isolated by flash chromatography on silica gel (*n*-hexane : ethyl acetate – 2 : 1) as colorless oil in 85% yield. ^1H NMR (700 MHz, Chloroform-*d*) δ 8.60 (ddd, *J* = 4.8, 1.8, 0.9 Hz, 1H), 7.58 (td, *J* = 7.6, 1.8 Hz, 1H), 7.32 (dd, *J* = 7.9, 1.6 Hz, 1H), 7.18 (ddd, *J* = 7.5, 4.9, 1.1 Hz, 1H), 6.98 – 6.95 (m, 2H), 6.92 (ddd, *J* = 7.6, 1.6, 0.6 Hz, 1H), 3.72 (dddd, *J* = 8.7, 7.0, 5.7, 3.7 Hz, 1H), 3.09 (dd, *J* = 13.8, 7.0 Hz, 1H), 2.95 (dd, *J* = 13.8, 8.7 Hz, 1H), 2.80 (dd, *J* = 16.0, 5.7 Hz, 1H), 2.76 (dd, *J* = 16.0, 3.7 Hz, 1H). ^{13}C NMR (176 MHz, Chloroform-*d*) δ 166.8, 157.6, 149.8, 147.4, 136.8, 129.4, 128.1, 126.2, 124.8, 124.4, 122.2, 122.1, 43.0, 35.7, 33.9. HRMS: Calculated for [$C_{15}H_{12}ClNO_2+H^+$]: 274.0629, found 274.0637.

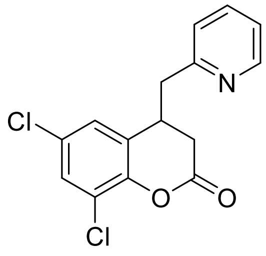

6,8-Dichloro-4-[(pyridin-2-yl)methyl]-3,4-dihydro-2*H*-1-benzopyran-2-one (4j) pure product was isolated by flash chromatography on silica gel (*n*-hexane : ethyl acetate – 2 : 1) as colorless oil in 47% yield. ^{1}H NMR (700 MHz, Chloroform-*d*) δ 8.60 (ddd, *J* = 4.8, 1.7, 0.9 Hz, 1H), 7.61 (td, *J* = 7.6, 1.8 Hz, 1H), 7.33 (d, *J* = 2.4 Hz, 1H), 7.20 (ddd, *J* = 7.5, 4.9, 1.0 Hz, 1H), 6.98 (d, *J* = 7.7 Hz, 1H), 6.93 (dd, *J* = 2.4, 0.6 Hz, 1H), 3.71 (dddd, *J* = 8.7, 6.9, 5.3, 4.1 Hz, 1H), 3.08 (dd, *J* = 13.9, 6.9 Hz, 1H), 2.94 (dd, *J* = 13.9, 8.7 Hz, 1H), 2.81 – 2.74 (m, 1H). ^{13}C NMR (176 MHz, Chloroform-*d*) δ 166.1, 157.1, 149.9, 146.3, 136.9, 129.5, 129.2, 129.2, 126.3, 124.3, 123.1, 122.3, 42.7, 35.6, 33.6. HRMS: Calculated for [$C_{15}H_{11}Cl_2NO_2$+H^+]: 308.0240, found 308.0247.

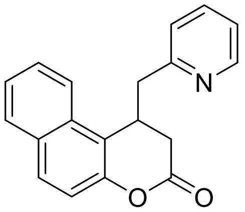

1-[(Pyridin-2-yl)methyl]-1*H*,2*H*,3*H*-naphtho[2,1-b]pyran-3-one (4k) pure product was isolated by flash chromatography on silica gel (*n*-hexane : ethyl acetate – 3 : 1) as colorless oil in 81% yield. ^{1}H NMR (700 MHz, Chloroform-*d*) δ 8.65 (ddd, *J* = 4.8, 1.8, 0.9 Hz, 1H), 8.00 (d, *J* = 8.4 Hz, 1H), 7.84 (d, *J* = 8.1 Hz, 1H), 7.79 (d, *J* = 8.8 Hz, 1H), 7.55 – 7.50 (m, 2H), 7.45 (ddd, *J* = 8.0, 6.8, 1.1 Hz, 1H), 7.26 (d, *J* = 8.8 Hz, 1H), 7.17 (ddd, *J* = 7.5, 4.9, 0.9 Hz, 1H), 6.97 (d, *J* = 7.7 Hz, 1H), 4.33 (dddd, *J* = 10.1, 6.3, 5.1, 1.8 Hz, 1H), 3.24 (dd, *J* = 14.0, 5.1 Hz, 1H), 2.92 (dd, *J* = 14.1, 10.1 Hz, 1H), 2.88 (dd, *J* = 16.1, 1.8 Hz, 1H), 2.81 (ddd, *J* = 16.1, 6.3, 0.8 Hz, 1H). ^{13}C NMR (176 MHz, Chloroform-*d*) δ 168.1, 158.0, 149.9, 149.2, 136.7, 131.1, 130.7, 129.4, 128.8, 127.4, 125.3, 124.5, 122.9, 122.0, 119.6, 117.6, 42.1, 33.3, 31.9. HRMS: Calculated for [$C_{19}H_{15}NO_2$+H^+]: 290.1176, found 290.1185.

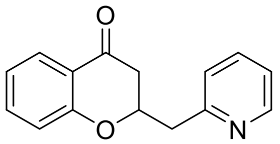

2-[(Pyridin-2-yl)methyl]-3,4-dihydro-2*H*-1-benzopyran-4-one (5a) pure product was isolated by flash chromatography on silica gel (*n*-hexane : ethyl acetate – 3 : 1) as yellow oil in 70% yield following regular procedure and in 65% yield following ultrasound procedure. ^{1}H NMR (700 MHz, Chloroform-*d*) δ 8.56 (ddd, *J* = 4.9, 1.8, 0.9 Hz, 1H), 7.88 (ddd, *J* = 7.8, 1.8, 0.4 Hz, 1H), 7.65 (td, *J* = 7.7, 1.8 Hz, 1H), 7.45 (ddd, *J* = 8.4, 7.2, 1.8 Hz, 1H), 7.28 – 7.25 (m, 1H), 7.19 (ddd, *J* = 7.5, 4.9, 1.1 Hz, 1H), 7.00 (ddd, *J* = 7.9, 7.2, 1.1 Hz, 1H), 6.93 (ddd, *J* = 8.4, 1.0, 0.4 Hz, 1H), 4.96 (dddd, *J* = 9.7, 7.0, 6.0, 5.7 Hz, 1H), 3.39 (dd, *J* = 13.9, 7.0 Hz, 1H), 3.20 (dd, *J* = 13.9, 6.0 Hz, 1H), 2.79 – 2.73 (m, 2H). ^{13}C NMR (176 MHz, Chloroform-*d*) δ 192.2, 161.5, 156.8, 149.7, 136.6, 136.1, 127.1, 124.4, 122.1, 121.5, 121.2, 118.1, 77.5, 43.5, 42.7. HRMS: Calculated for [$C_{15}H_{13}NO_2$+H^+]: 240.1019, found 240.1029.

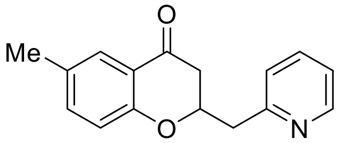

6-Methyl-2-[(pyridin-2-yl)methyl]-3,4-dihydro-2*H*-1-benzopyran-4-one (5b) pure product was isolated by flash chromatography on silica gel (*n*-hexane : ethyl acetate – 3 : 1) as yellow oil in 53% yield. ^{1}H NMR (700 MHz, Chloroform-*d*) δ 8.56 (dd, *J* = 4.8, 1.7 Hz, 1H), 7.68 – 7.62 (m, 2H), 7.27 – 7.25 (m, 2H), 7.18 (ddd, *J* = 7.6, 4.9, 1.2 Hz, 1H), 6.83 (d, *J* = 8.5 Hz, 1H), 4.91 (ddt, *J* = 9.2, 7.0, 6.1 Hz, 1H), 3.37 (dd, *J* = 13.9, 7.0 Hz, 1H), 3.18 (dd, *J* = 13.9, 6.0 Hz, 1H), 2.76 – 2.70 (m, 2H), 2.29 (s, 3H). ^{13}C NMR (176 MHz, Chloroform-*d*) δ 192.5, 159.6, 157.0, 149.7, 137.2, 136.6, 130.9, 126.7, 124.4, 122.0, 120.8, 117.9, 77.4, 43.6, 42.7, 20.5. HRMS: Calculated for [$C_{16}H_{15}NO_2$+H^+]: 254.1176, found 254.1189.

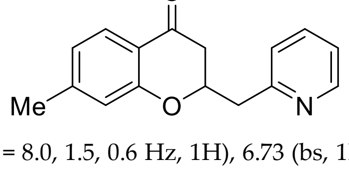

7-Methyl-2-[(pyridin-2-yl)methyl]-3,4-dihydro-2*H*-1-benzopyran-4-one (5c) pure product was isolated by flash chromatography on silica gel (*n*-hexane : ethyl acetate – 3 : 1) as yellow oil in 54% yield. ^{1}H NMR (700 MHz, Chloroform-*d*) δ 8.56 (ddd, *J* = 4.9, 1.8, 0.9 Hz, 1H), 7.76 (d, *J* = 8.0 Hz, 1H), 7.65 (td, *J* = 7.7, 1.8 Hz, 1H), 7.26 (d, *J* = 7.8 Hz, 1H), 7.18 (ddd, *J* = 7.5, 4.9, 1.1 Hz, 1H), 6.81 (ddd, *J* = 8.0, 1.5, 0.6 Hz, 1H), 6.73 (bs, 1H), 4.92 (dtd, *J* = 8.1, 7.1, 6.0 Hz, 1H), 3.36 (dd, *J* = 13.9, 7.1 Hz, 1H), 3.18 (dd, *J* = 13.9, 5.9 Hz, 1H), 2.75 – 2.67 (m, 2H), 2.33 (s, 3H). ^{13}C NMR (176 MHz, Chloroform-*d*) δ 191.9, 161.6, 157.0, 149.7, 147.6, 136.6, 127.0, 124.4, 122.9, 122.0, 119.0, 118.1, 77.5, 43.6, 42.6, 22.0. HRMS: Calculated for [$C_{16}H_{15}NO_2$+H^+]: 254.1176, found 254.1183.

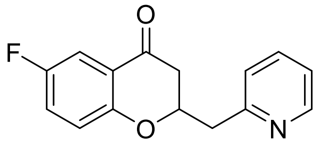

6-Fluoro-2-[(pyridin-2-yl)methyl]-3,4-dihydro-2*H*-1-benzopyran-4-one (5d) pure product was isolated by flash chromatography on silica gel (*n*-hexane : ethyl acetate – 3 : 1) as colorless oil in 74%. ^{1}H NMR (700 MHz, Chloroform-*d*) δ 8.55 (ddd, *J* = 4.9, 1.7, 0.9 Hz, 1H), 7.66 (td, *J* = 7.7, 1.8 Hz, 1H), 7.51 (dd, *J* = 8.2, 3.2 Hz, 1H), 7.25 (d, *J* = 7.0 Hz, 1H), 7.19 (ddd, *J* = 7.5, 4.9, 1.1 Hz, 1H), 7.16 (ddd, *J* = 9.0, 7.7, 3.2 Hz, 1H), 6.90 (dd, *J* = 9.1, 4.2 Hz, 1H), 4.93 (dddd, *J* = 11.6, 6.9, 6.0, 3.9 Hz, 1H), 3.37 (dd, *J* = 14.0, 6.9 Hz, 1H), 3.19 (dd, *J* = 14.0, 6.0 Hz, 1H), 2.77 (dd, *J* = 16.9, 3.9 Hz, 1H), 2.73 (dd, *J* = 16.9, 11.7 Hz, 1H). ^{13}C NMR (176 MHz,

Chloroform-*d*) δ 191.4 (d, *J* = 1.6 Hz), 157.8 (d, *J* = 1.6 Hz), 157.3 (d, *J* = 241.1 Hz), 156.6, 149.7, 136.7, 124.3, 123.6 (d, *J* = 24.8 Hz), 122.1, 121.6 (d, *J* = 6.5 Hz), 119.7 (d, *J* = 9.5 Hz), 112.0 (d, *J* = 23.5 Hz), 77.7, 43.4, 42.4. HRMS: Calculated for [$C_{15}H_{12}FNO_2$+H^+]: 258.0925, found 258.0931.

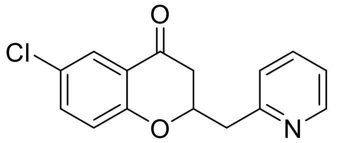

6-Chloro-2-[(pyridin-2-yl)methyl]-3,4-dihydro-2*H*-1-benzopyran-4-one (5e) pure product was isolated by flash chromatography on silica gel (*n*-hexane : ethyl acetate – 2 : 1) as yellow oil in 41% yield following regular procedure. The reaction did not yield desired product following ultrasound procedure. ^{1}H NMR (700 MHz, Chloroform-*d*) δ 8.56 (d, *J* = 5.3 Hz, 1H), 7.82 (d, *J* = 2.6 Hz, 1H), 7.66 (td, *J* = 7.7, 1.8 Hz, 1H), 7.38 (dd, *J* = 8.8, 2.7 Hz, 1H), 7.25 (d, *J* = 7.0 Hz, 1H), 7.20 (dd, *J* = 7.8, 4.6 Hz, 1H), 6.89 (d, *J* = 8.8 Hz, 1H), 4.95 (dddd, *J* = 11.8, 6.9, 6.0, 3.8 Hz, 1H), 3.37 (dd, *J* = 14.0, 6.9 Hz, 1H), 3.20 (dd, *J* = 14.0, 6.1 Hz, 1H), 2.78 (dd, *J* = 16.9, 3.9 Hz, 1H), 2.74 (dd, *J* = 16.9, 11.8 Hz, 1H). ^{13}C NMR (176 MHz, Chloroform-*d*) δ 191.0, 160.0, 156.6, 149.8, 136.7, 135.9, 127.1, 126.5, 124.3, 122.2, 122.0, 119.9, 77.8, 43.4, 42.4. HRMS: Calculated for [$C_{15}H_{12}ClNO_2$+H^+]: 274.0629, found 274.0635.

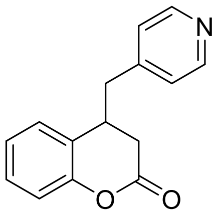

4-[(Pyridin-4-yl)methyl]-3,4-dihydro-2*H*-1-benzopyran-2-one (7) pure product was isolated by flash chromatography on silica gel (*n*-hexane : ethyl acetate – 3 : 1) as colorless oil in over 95% yield following ultrasound procedure. Regular procedure did not yield desired product. ^{1}H NMR (700 MHz, Chloroform-*d*) δ 8.52 – 8.51 (m, 2H), 7.28 (ddd, *J* = 8.1, 7.6, 1.6 Hz, 1H), 7.08 (dd, *J* = 8.1, 1.1 Hz, 1H), 7.04 (td, *J* = 7.5, 1.2 Hz, 1H), 7.01 – 6.98 (m, 2H), 6.93 (dd, *J* = 7.5, 1.6 Hz, 1H), 3.26 (dddd, *J* = 8.5, 7.4, 5.8, 3.5 Hz, 1H), 2.91 (dd, *J* = 13.6, 7.4 Hz, 1H), 2.81 – 2.75 (m, 2H), 2.73 (dd, *J* = 16.0, 3.5 Hz, 1H). ^{13}C NMR (176 MHz, Chloroform-*d*) δ 167.5, 151.4, 150.2, 146.9, 129.0, 127.9, 125.1, 124.6, 124.6, 117.4, 40.7, 36.8, 34.2. HRMS: Calculated for [$C_{15}H_{13}NO_2$+H^+]: 240.1019, found 240.1028.

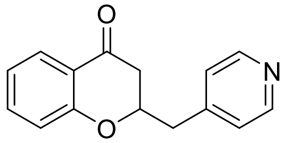

2-[(Pyridin-4-yl)methyl]-3,4-dihydro-2*H*-1-benzopyran-4-one (8) pure product was isolated by flash chromatography on silica gel (*n*-hexane : ethyl acetate – 3 : 1) as yellow oil in 23% yield following regular procedure and in 75% yield following ultrasound procedure. ^{1}H NMR (700 MHz, Chloroform-*d*) δ 8.58 – 8.52 (m, 2H), 7.86 (ddd, *J* = 7.8, 1.8, 0.4 Hz, 1H), 7.47 (ddd, *J* = 8.4, 7.2, 1.8 Hz, 1H), 7.24 – 7.18 (m, 2H), 7.02 (ddd, *J* = 7.9, 7.2, 1.1 Hz, 1H), 6.94 (ddd, *J* = 8.4, 1.0, 0.4 Hz, 1H), 4.71 (dddd, *J* = 9.6, 7.3, 5.9, 5.2 Hz, 1H), 3.16 (dd, *J* = 14.2, 7.4 Hz, 1H), 3.04 (dd, *J* = 14.2, 5.1 Hz, 1H), 2.74 – 2.67 (m, 2H). ^{13}C NMR (176 MHz, Chloroform-*d*) δ 191.6, 161.2, 150.2 (2C), 145.5, 136.3, 127.2, 125.0 (2C), 121.8, 121.1, 118.1, 77.3, 42.6, 40.6. HRMS: Calculated for [$C_{15}H_{13}NO_2$+H^+]: 240.1019, found 240.1026.

3. **Enantioselective synthesis of 2-[(pyridin-2-yl)methyl]-3,4-dihydro-2*H*-1-benzopyran-4-one 5a**

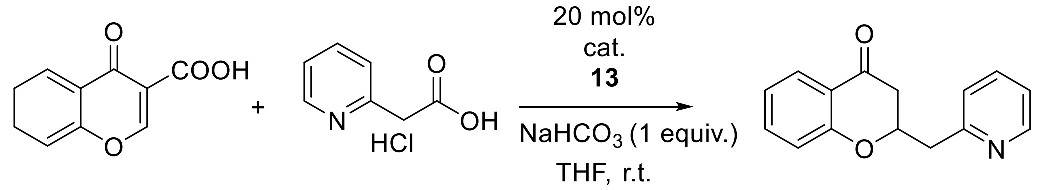

3a **1** **5a**

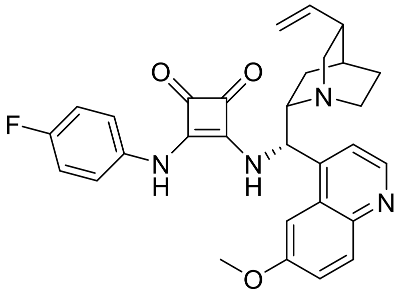

13a
4% *ee*
yield: 46%

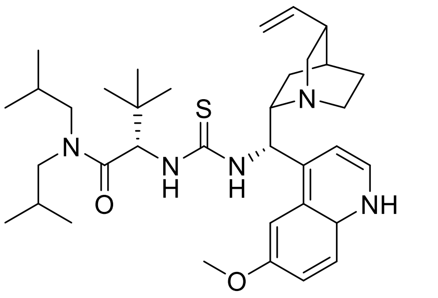

13b
3% *ee*
yield: 42%

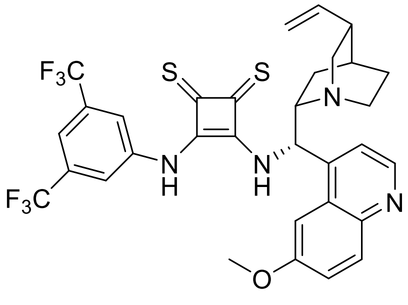

13c
0% *ee*
yield: 46%

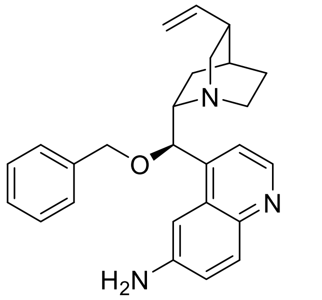

13d
9% *ee*
yield: 40%

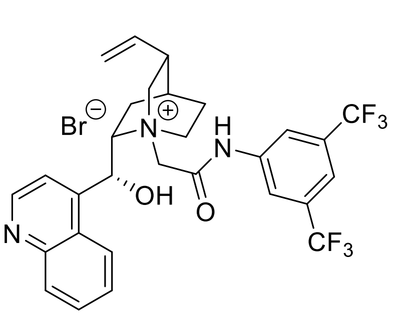

13e
8% *ee*
yield: 40%

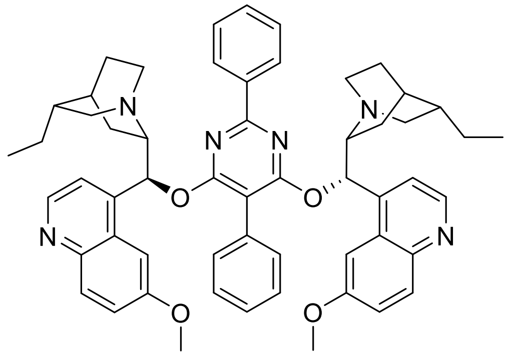

13f
14% *ee*
yield: 54%

An ordinary screw-cap vial was charged with a magnetic stirring bar, chromone-3-carboxylic acids **3a** (0.1 mmol, 1 equivalent), THF (0.2 mL), catalyst **13** 20 mol% and $NaHCO_3$ (0.1 mmol, 1 eqiuv.) to counterbalance the hydrochloride **1a** (0.1 mmol, 1.0 equivalent.)The reaction mixture was stirred at room temperature and monitored by ^{1}H NMR spectroscopy. After the complete consumption of the carboxylic acid **4**, the mixture was directly subjected to FC on silica gel (*n*-hexane:ethyl acetate 3:1) to afford pure product **5a**.

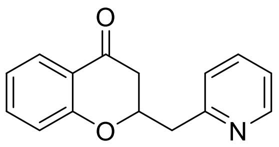

2-[(Pyridin-2-yl)methyl]-3,4-dihydro-2*H*-1-benzopyran-4-one (5a) pure product was isolated by flash chromatography on silica gel (hexane : ethyl acetate – 3 : 1) as yellow oil. ^{1}H NMR (700 MHz, Chloroform-*d*) δ 8.56 (ddd, *J* = 4.9, 1.8, 0.9 Hz, 1H), 7.88 (ddd, *J* = 7.8, 1.8, 0.4 Hz, 1H), 7.65 (td, *J* = 7.7, 1.8 Hz, 1H), 7.45 (ddd, *J* = 8.4, 7.2, 1.8 Hz, 1H), 7.28 – 7.25 (m, 1H), 7.19 (ddd, *J* = 7.5, 4.9, 1.1 Hz, 1H), 7.00 (ddd, *J* = 7.9, 7.2, 1.1 Hz, 1H), 6.93 (ddd, *J* = 8.4, 1.0, 0.4 Hz, 1H), 4.96 (dddd, *J* = 9.7, 7.0, 6.0, 5.7 Hz, 1H), 3.39 (dd, *J* = 13.9, 7.0 Hz, 1H), 3.20 (dd, *J* = 13.9, 6.0 Hz, 1H), 2.79 – 2.73 (m, 2H). ^{13}C NMR (176 MHz, Chloroform-*d*) δ 192.2, 161.5, 156.8, 149.7, 136.6, 136.1, 127.1, 124.4, 122.1, 121.5, 121.2, 118.1, 77.5, 43.5, 42.7. HRMS: Calculated for [$C_{15}H_{13}NO_2$+H^+]: 240.1019, found 240.1029. The er was determined by HPLC using a Chiralpak IA column [hexane/i-PrOH (90:10)]; flow rate 1.0 mL/min; τ_{major} =12.10 min; τ_{minor} = 13.24 min.

4. **Mechanistic considerations using MS analysis**

In order to clarify the assumed reaction mechanism, MS analysis was performed. An ordinary screw-cap vial was charged with a magnetic stirring bar, the chromone-3-carboxylic acids **3a** (0.1 mmol, 1 equivalent), THF (0.2 mL), *N*-methyl morpholine (0.17 mmol, 1.7 equivalent), and the pyridylacetic acid hydrochloride **1** (0.15 mmol, 1.5 equivalent). The reaction mixture was stirred at room temperature for 2 hours, the sample was taken (10 μL), diluted with MeOH (1 mL) and subjected to MS analysis [cationic mode, calculated for $C_{16}H_{13}NO_4$ [M+H]+: 284, found: 284].

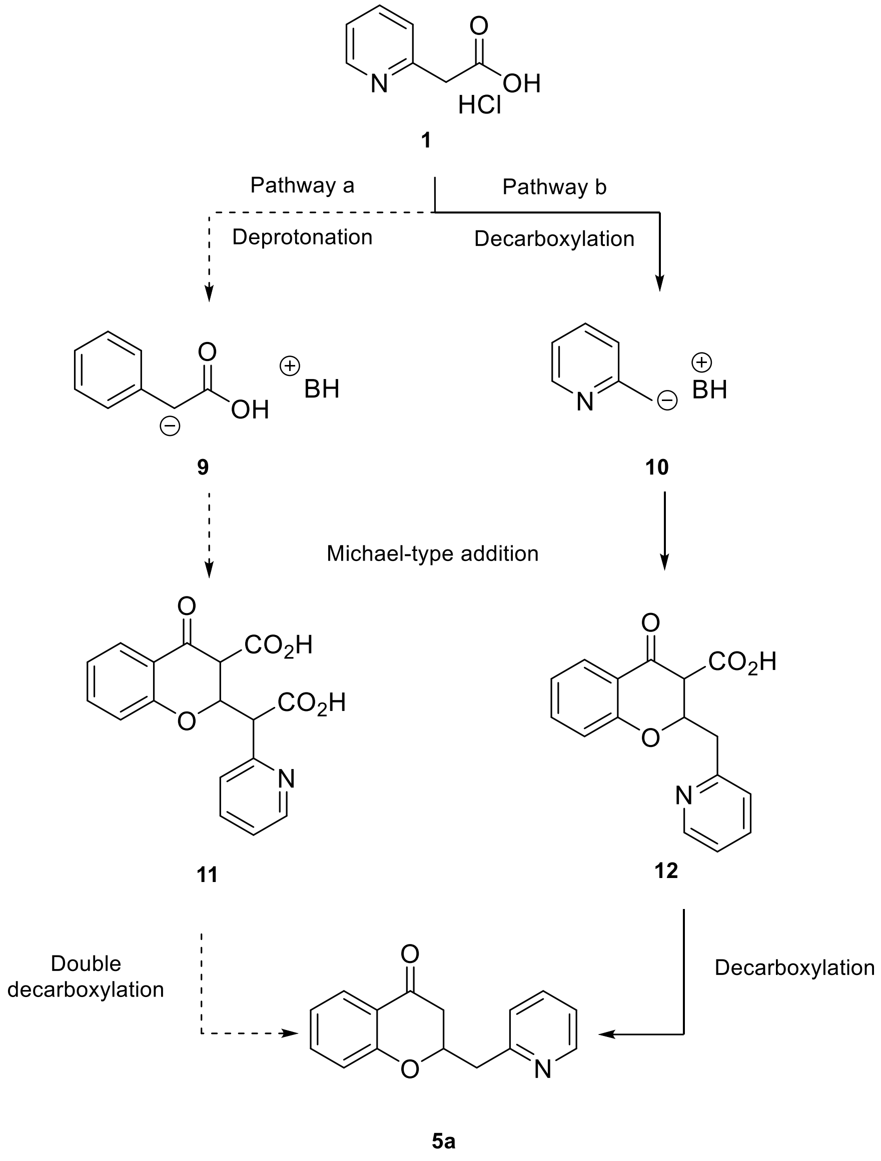

Start Ion: 50
End Ion: 500
Source: ESI + 3.5kV 350C
Capillary: 180V 300C Offset: 30V Span: 20V

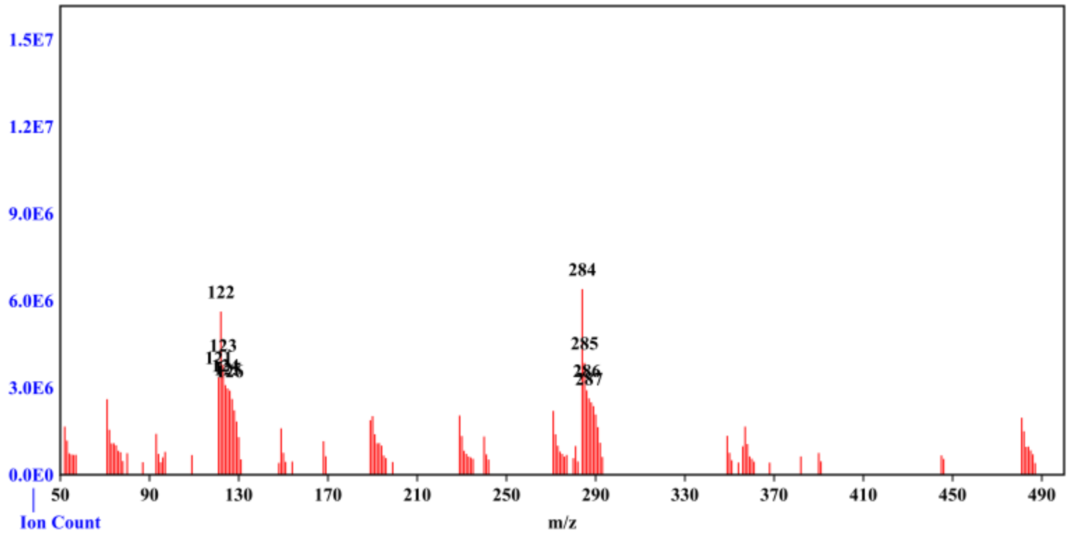

5. NMR spectra

4-[(Pyridin-2-yl)methyl]-3,4-dihydro-2*H*-1-benzopyran-2-one (4a)

^{1}H NMR

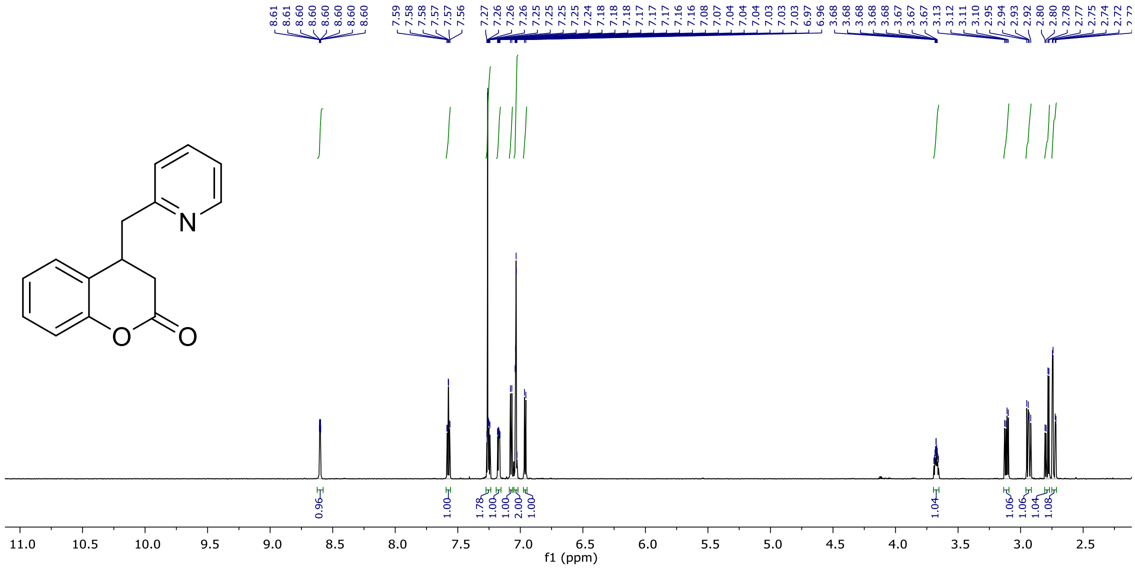

^{13}C NMR

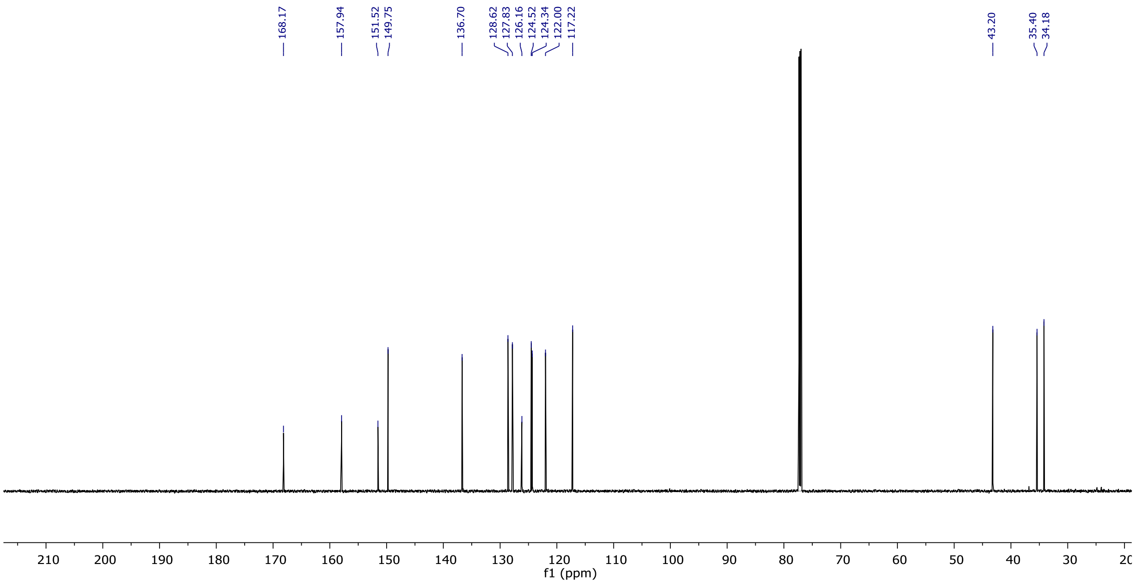

7-Methoxy-4-[(pyridin-2-yl)methyl]-3,4-dihydro-2*H*-1-benzopyran-2-one (4b)

^{1}H NMR

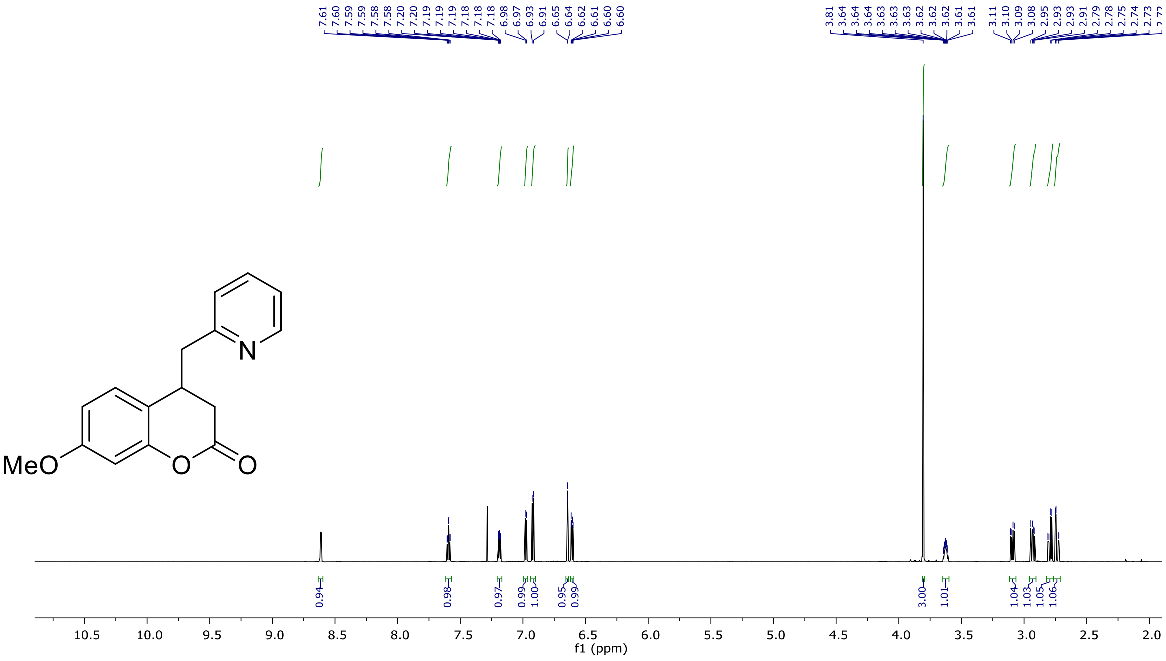

^{13}C NMR

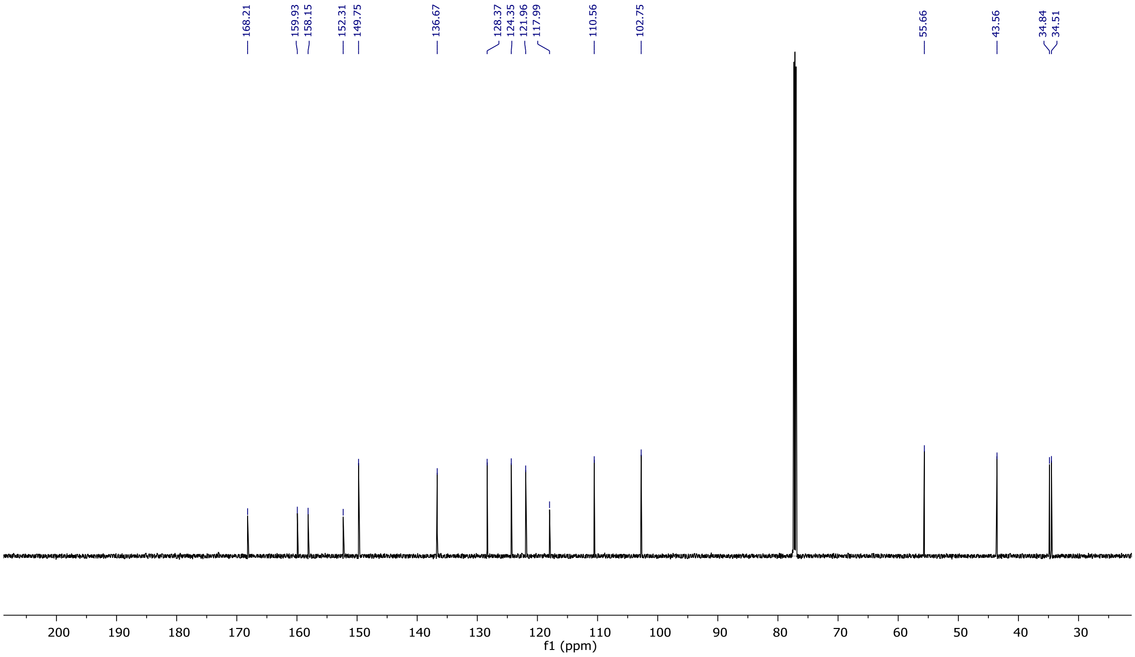

6-Methoxy-4-[(pyridin-2-yl)methyl]-3,4-dihydro-2*H*-1-benzopyran-2-one (4c)

[1] H NMR

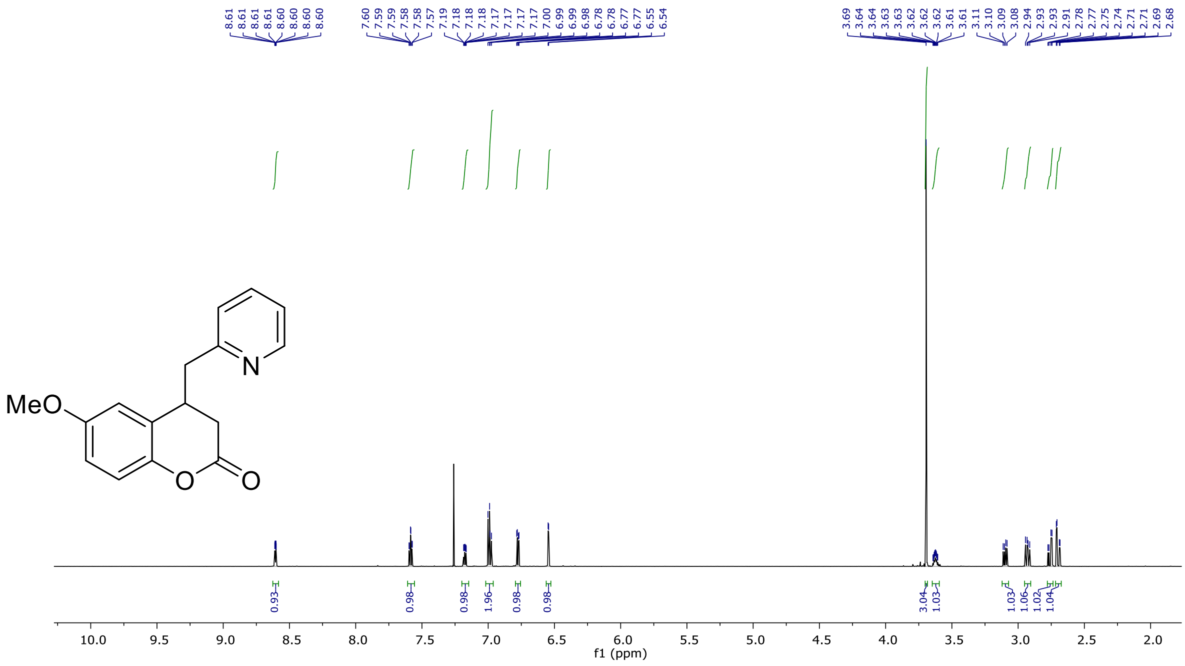

[13] C NMR

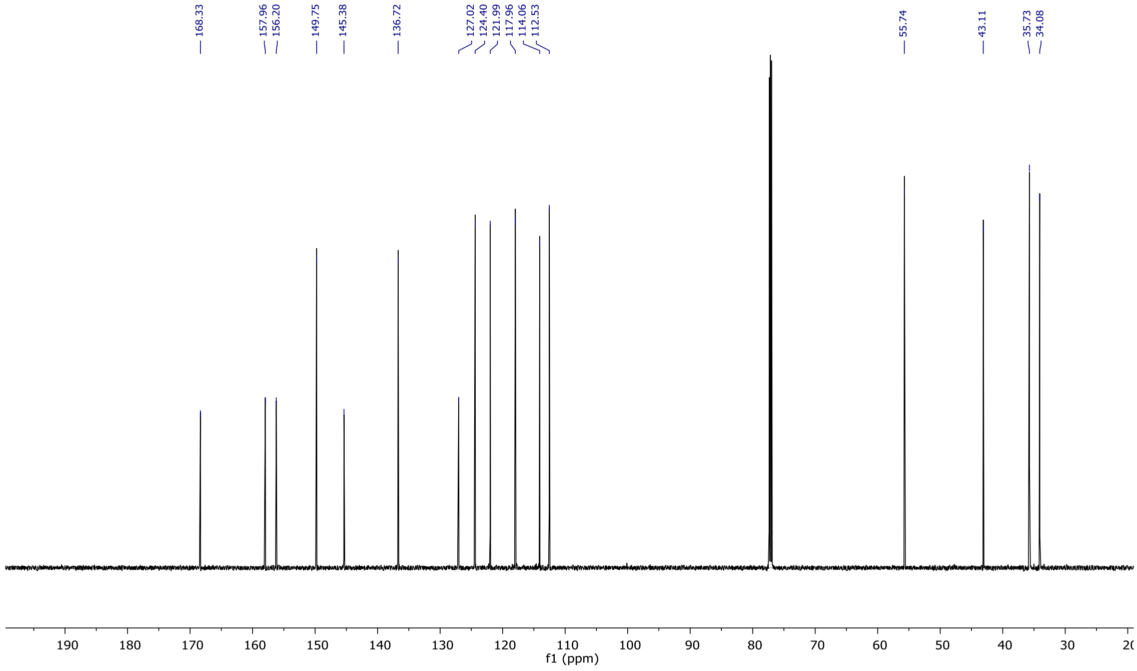

6-Nitro-4-[(pyridin-2-yl)methyl]-3,4-dihydro-2*H*-1-benzopyran-2-one (4d)

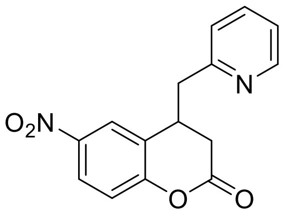

^{1}H NMR

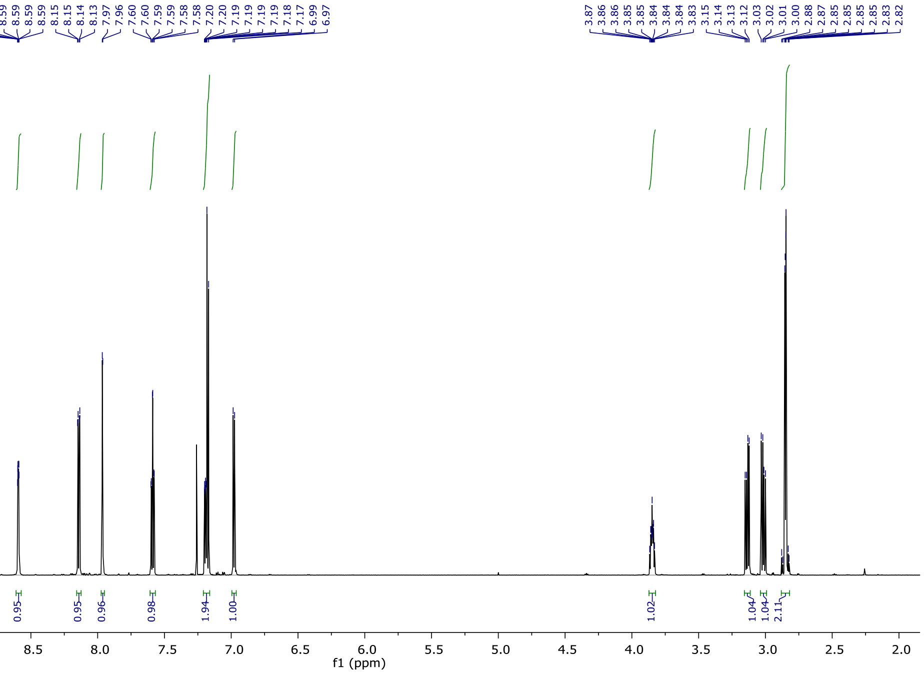

^{13}C NMR

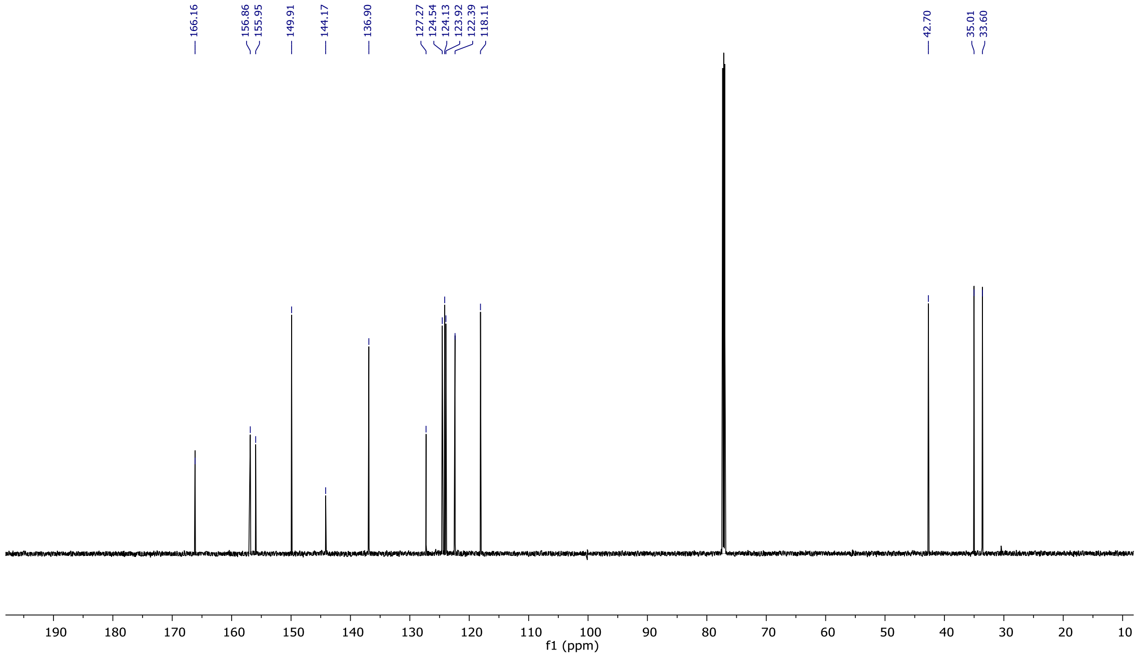

6-Fluoro-4-[(pyridin-2-yl)methyl]-3,4-dihydro-2*H*-1-benzopyran-2-one (4e)

[1] H NMR

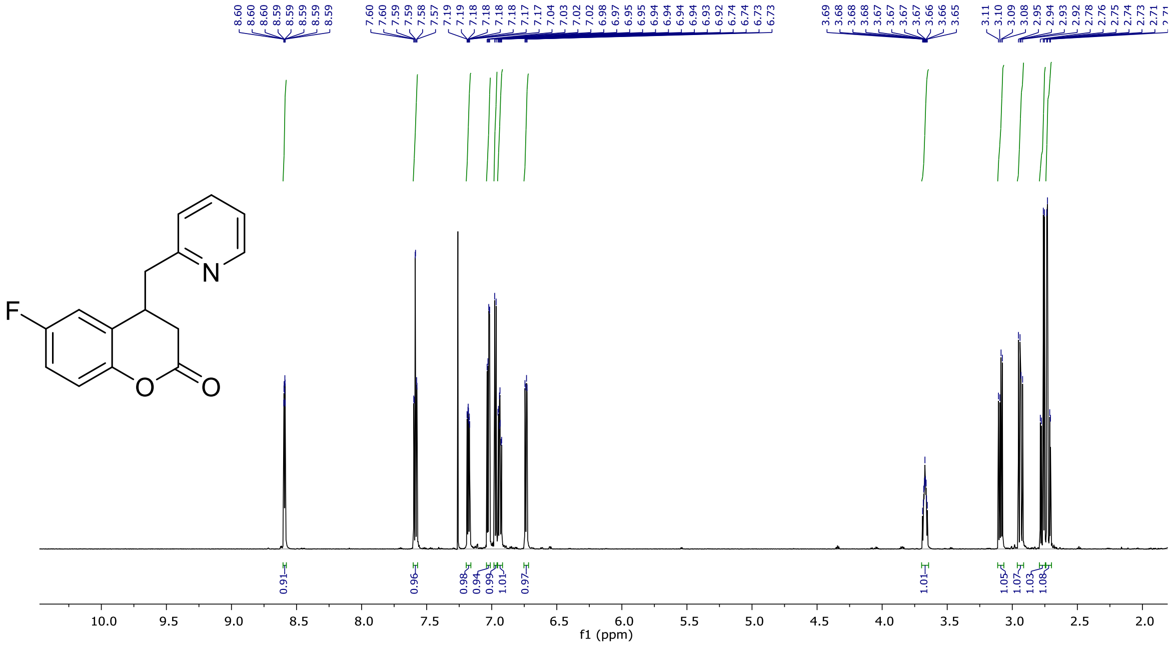

[13] C NMR

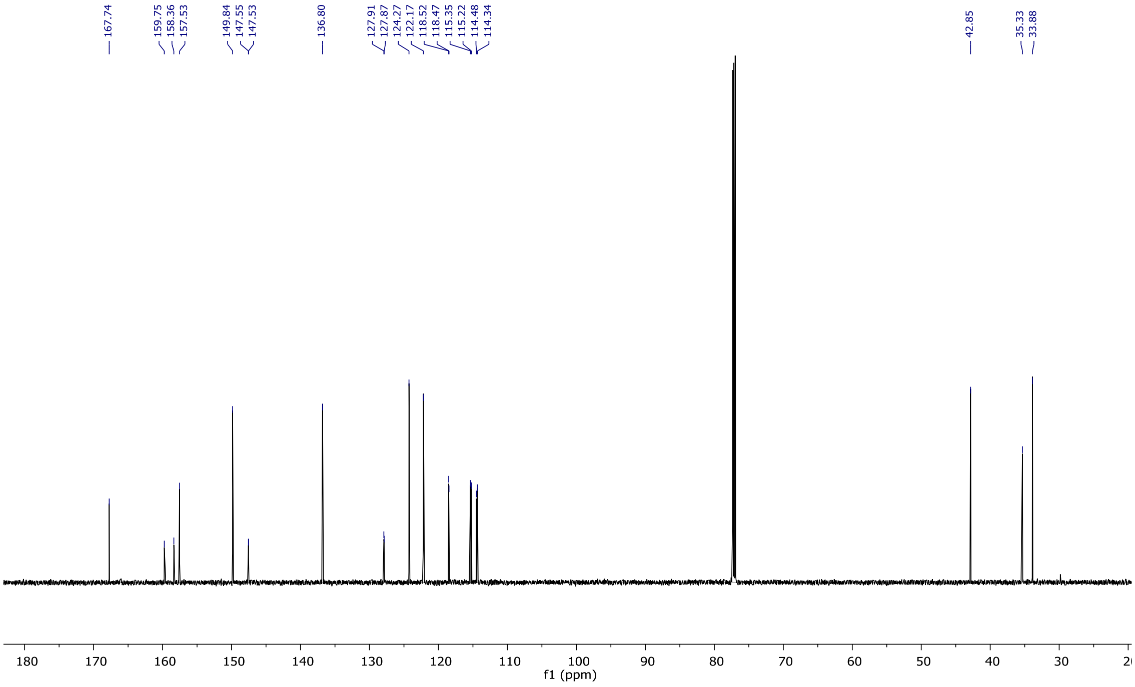

6-Chloro-4-[(pyridin-2-yl)methyl]-3,4-dihydro-2*H*-1-benzopyran-2-one (4f)

^{1}H NMR

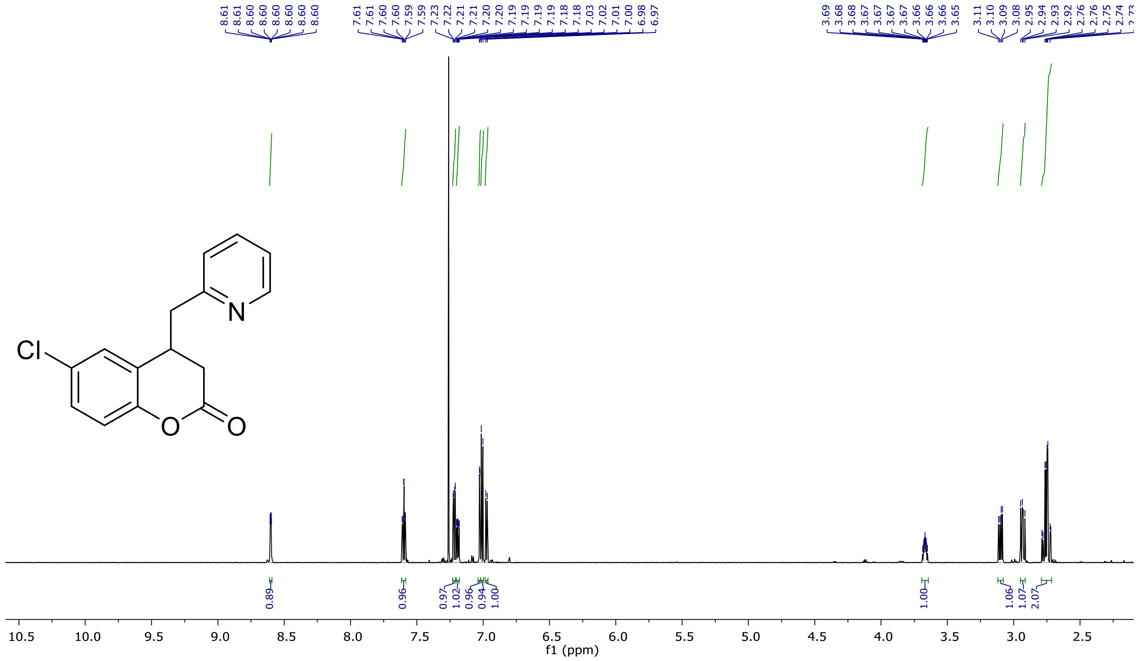

^{13}C NMR

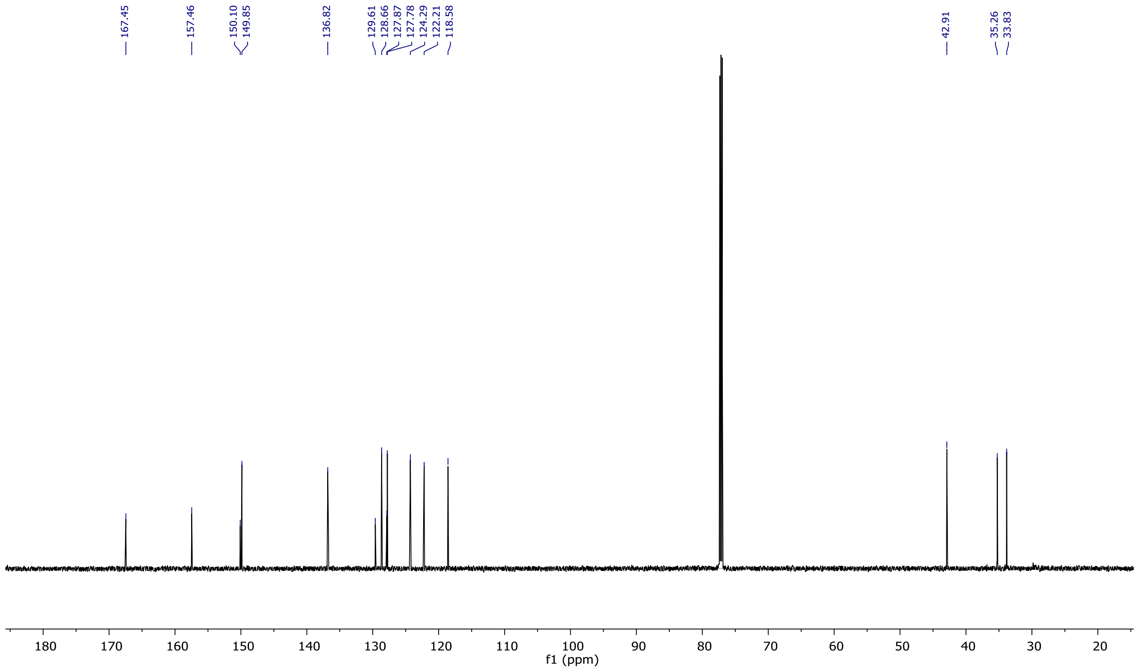

6-Bromo-4-[(pyridin-2-yl)methyl]-3,4-dihydro-2*H*-1-benzopyran-2-one (4g)

[1] H NMR

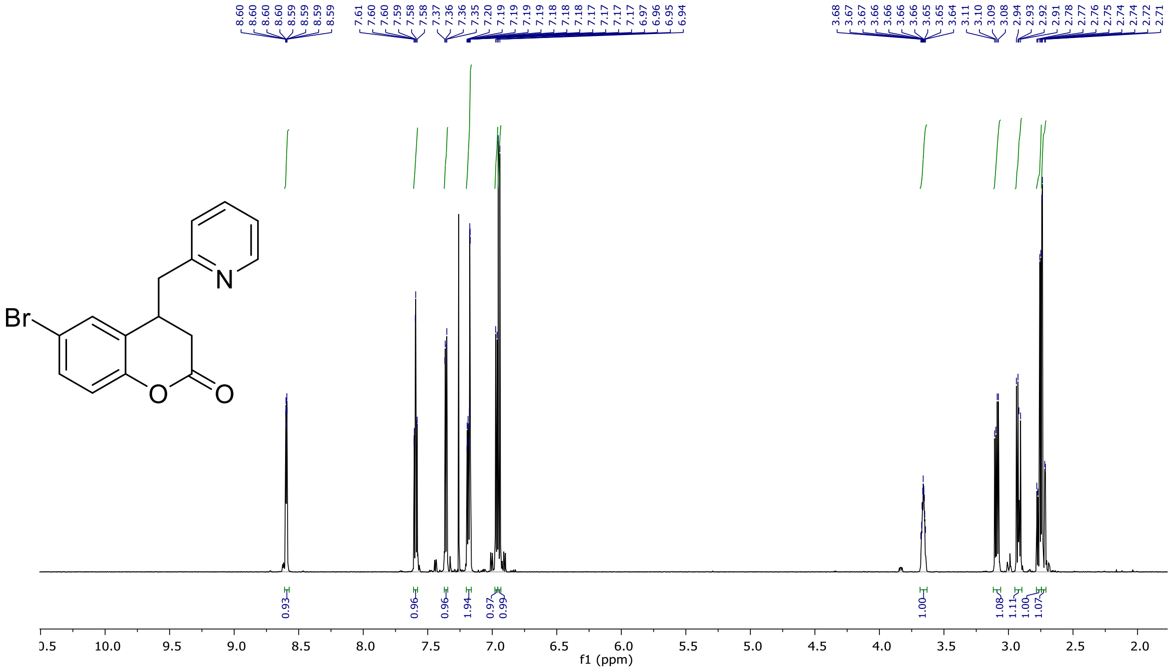

[13] C NMR

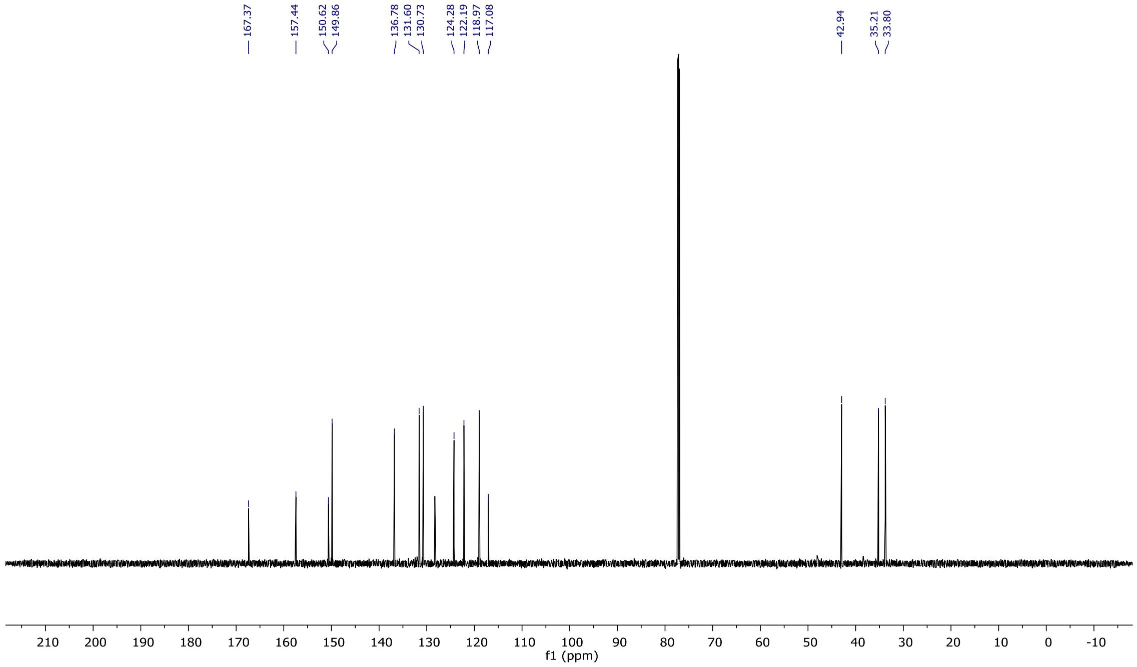

6-Methyl-4-[(pyridin-2-yl)methyl]-3,4-dihydro-2*H*-1-benzopyran-2-one (4h)

[1] H NMR

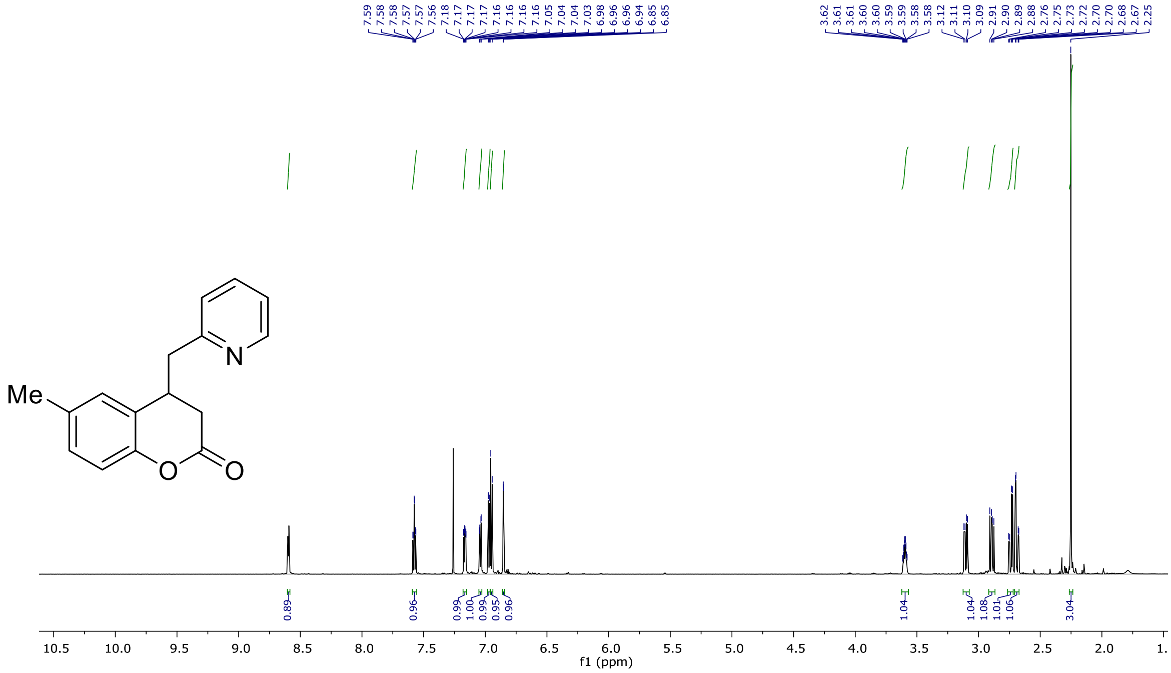

[13] C NMR

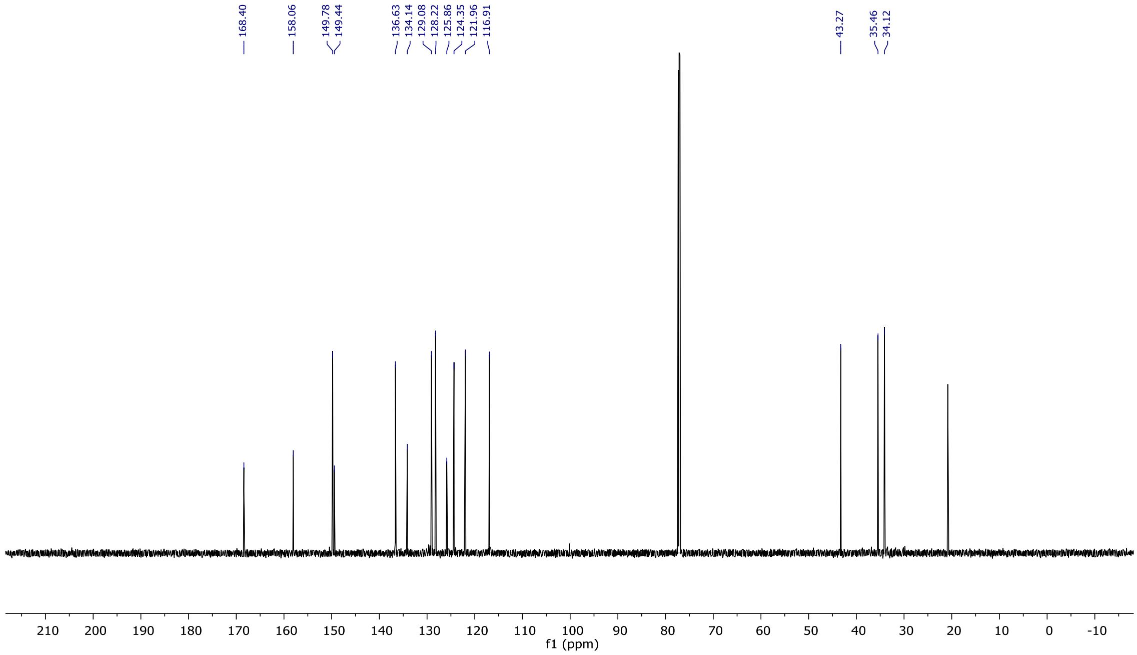

8-Chloro-4-[(pyridin-2-yl)methyl]-3,4-dihydro-2*H*-1-benzopyran-2-one (4i)

^{1}H NMR

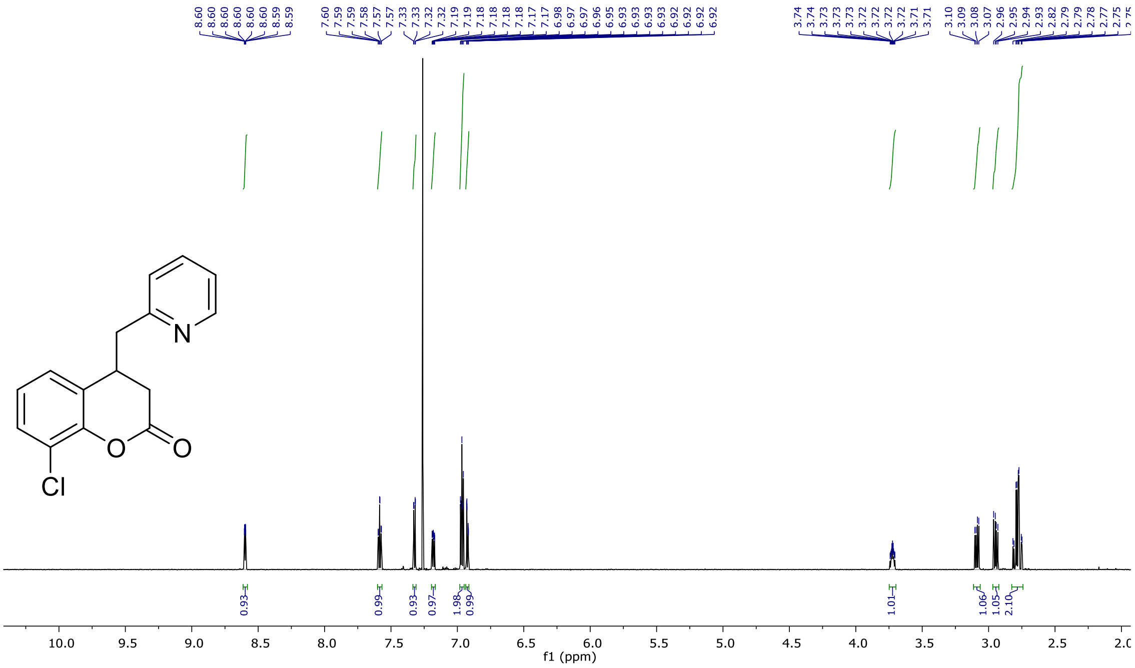

^{13}C NMR

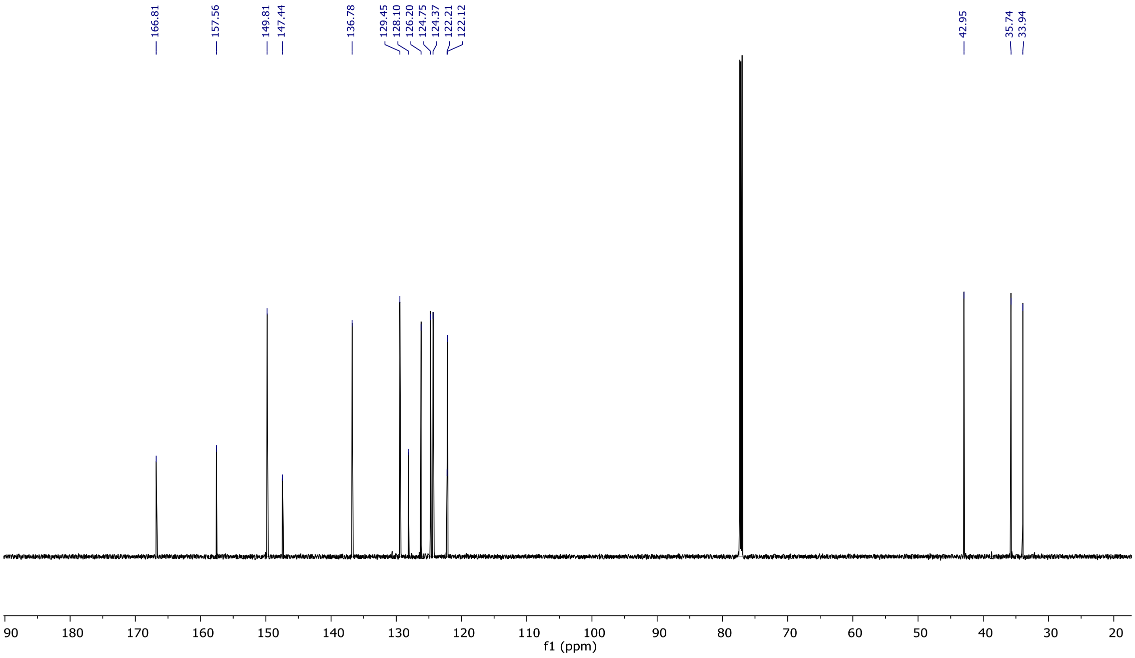

6,8-Dichloro-4-[(pyridin-2-yl)methyl]-3,4-dihydro-2*H*-1-benzopyran-2-one (4j)

^{1}H NMR

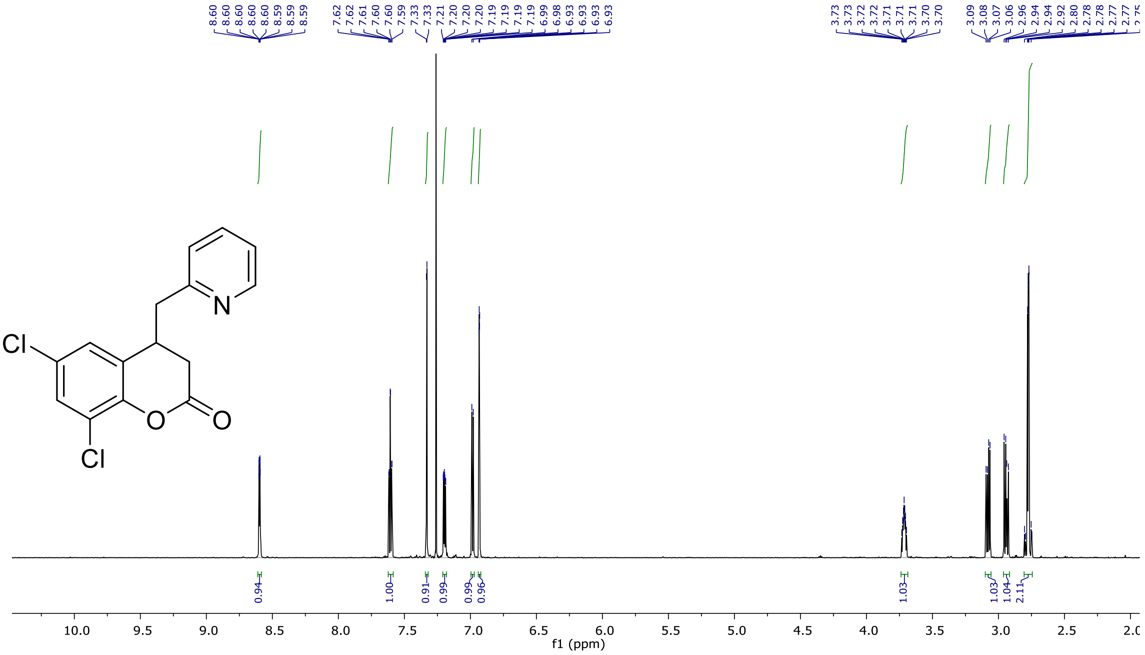

^{13}C NMR

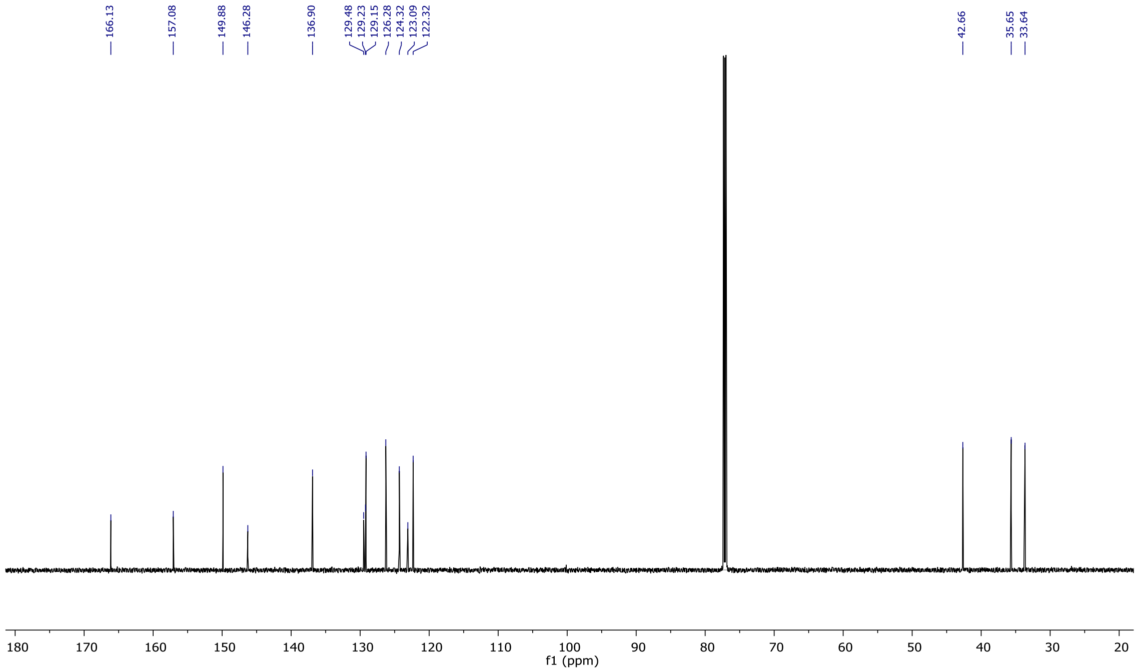

1-[(Pyridin-2-yl)methyl]-1*H*,2*H*,3*H*-naphtho[2,1-b]pyran-3-one (4k)

^{1} H NMR

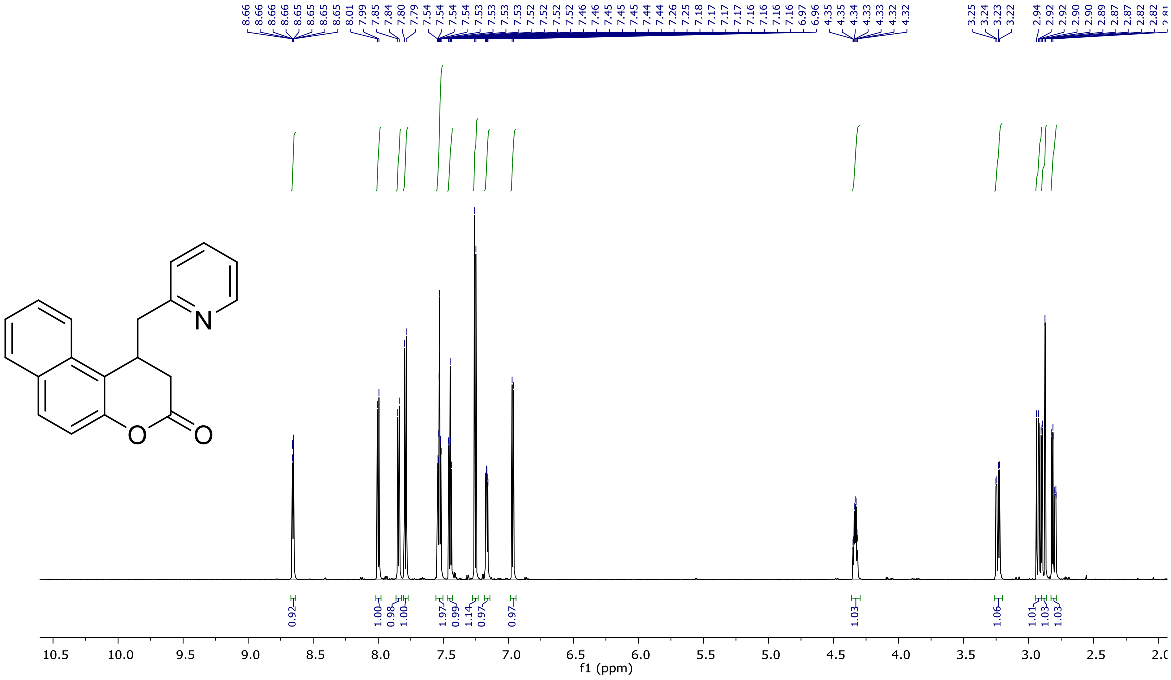

^{13} C NMR

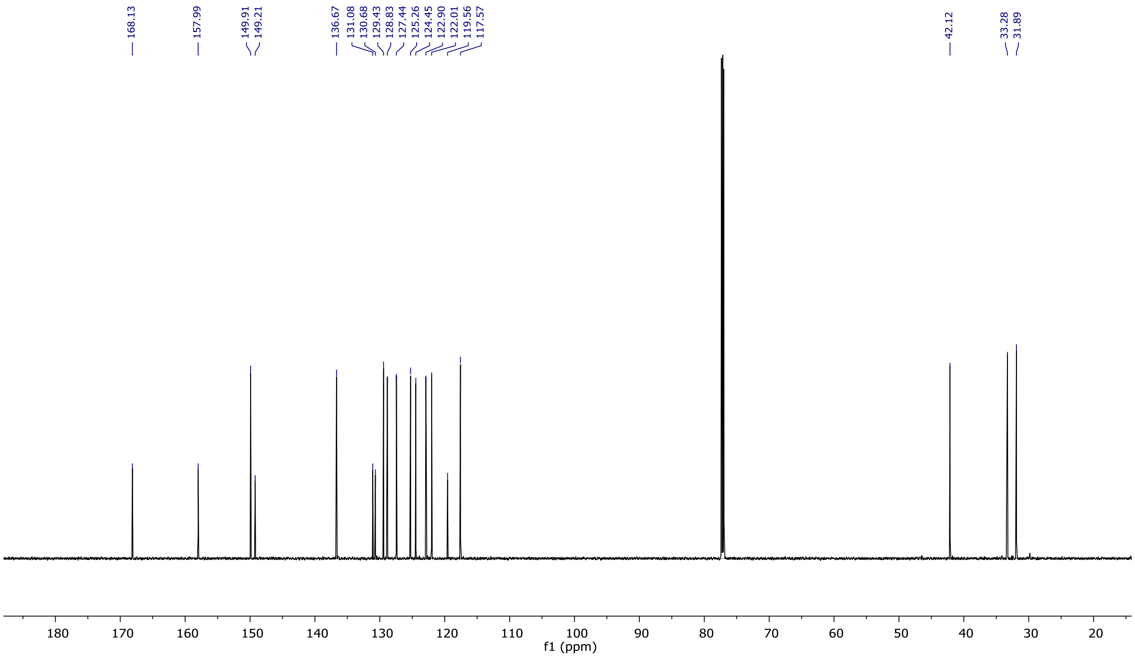

2-[(Pyridin-2-yl)methyl]-3,4-dihydro-2*H*-1-benzopyran-4-one (5a)

^{1}H NMR

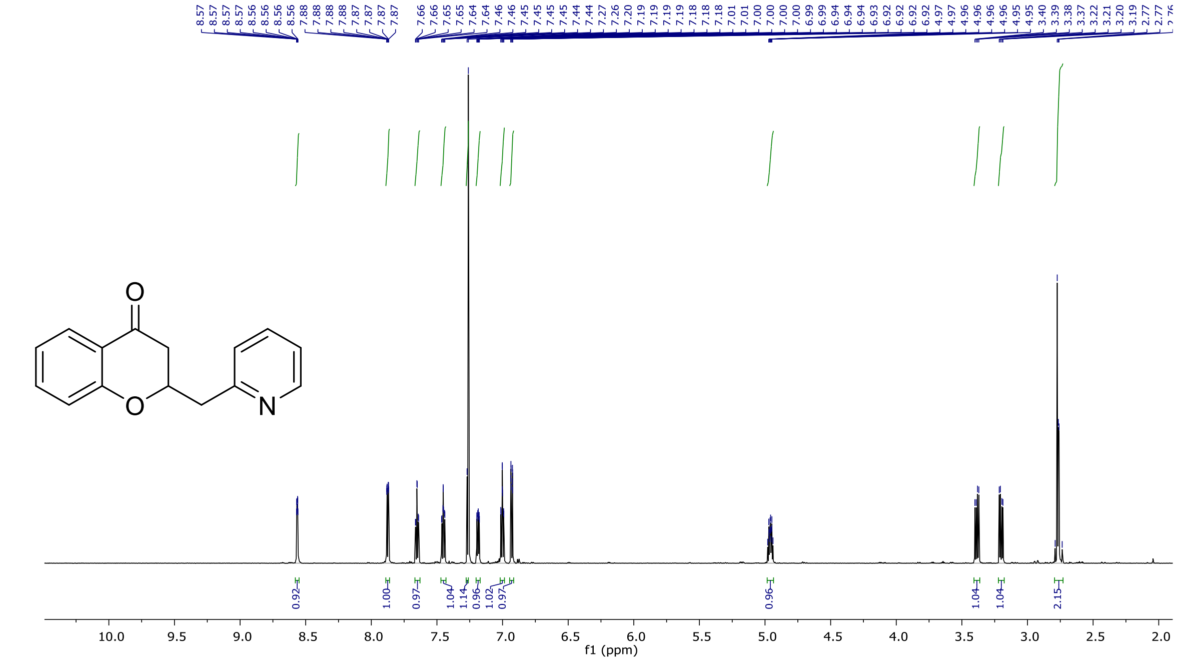

^{13}C NMR

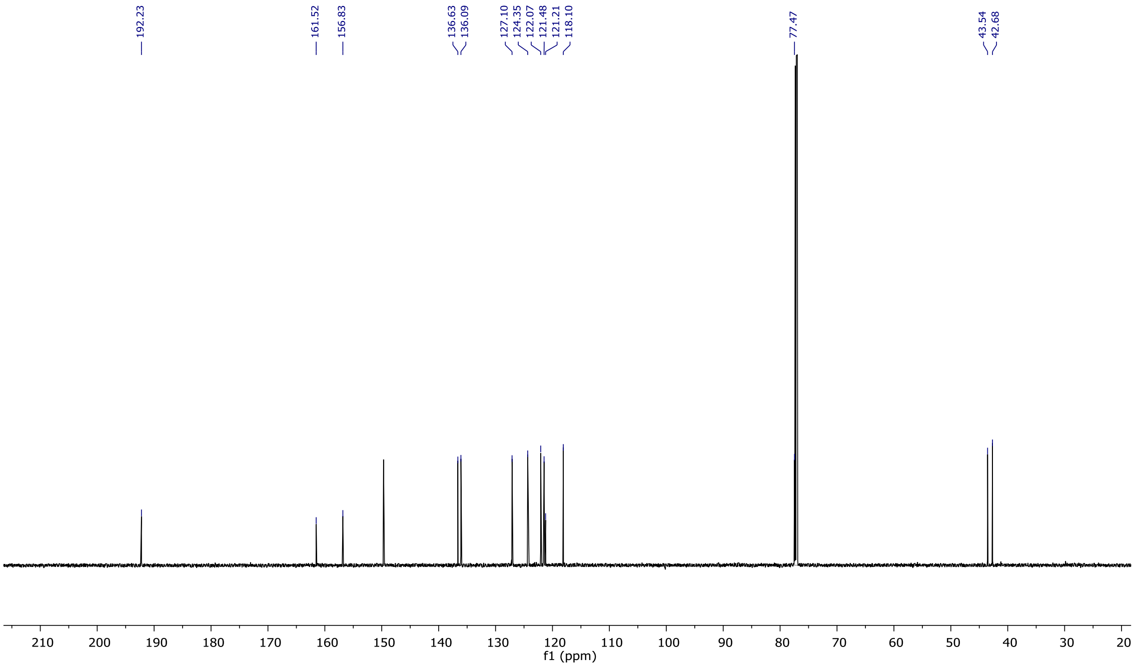

6-Methyl-2-[(pyridin-2-yl)methyl]-3,4-dihydro-2*H*-1-benzopyran-4-one (5b)

[1] H NMR

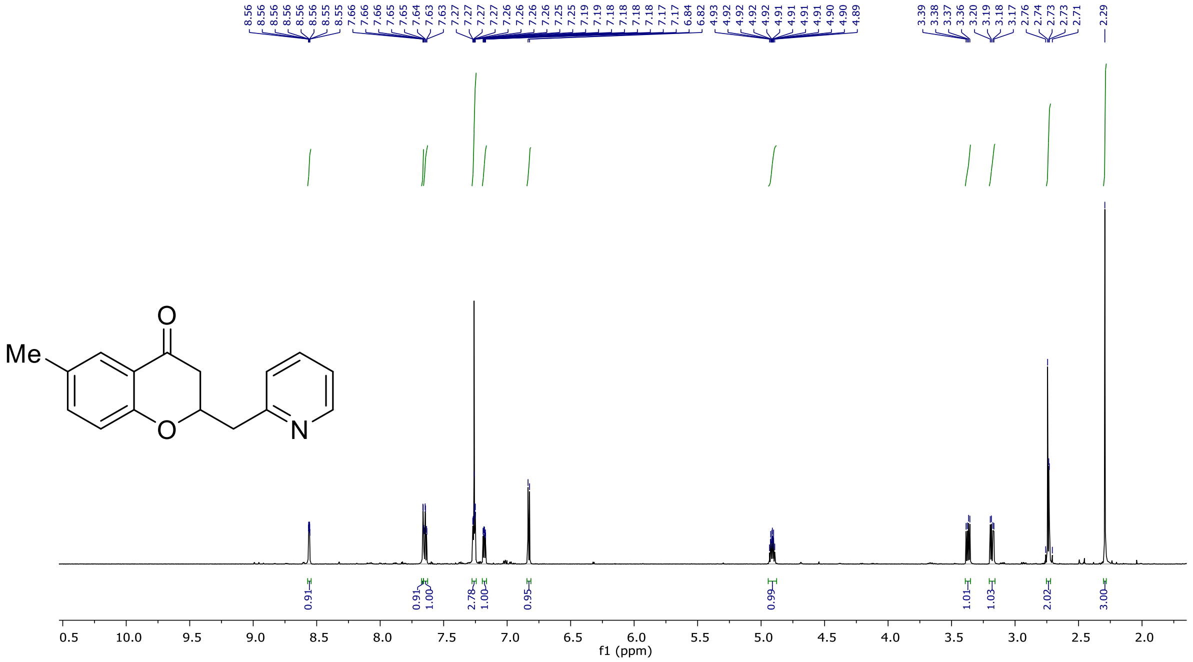

[13] C NMR

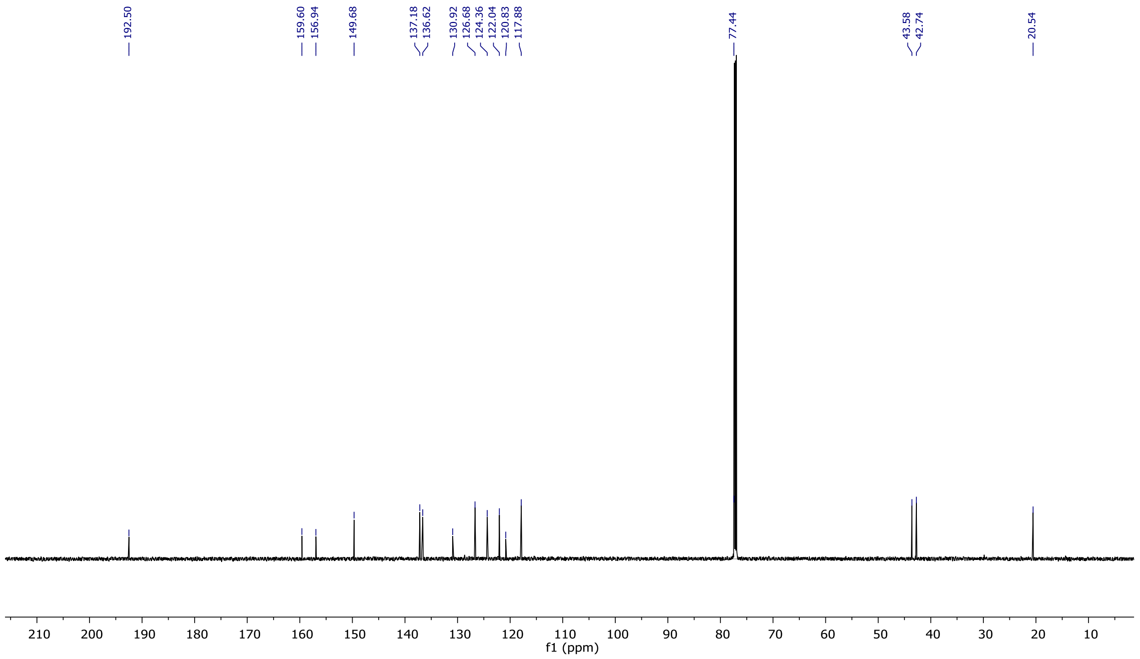

7-Methyl-2-[(pyridin-2-yl)methyl]-3,4-dihydro-2*H*-1-benzopyran-4-one (5c)

^{1}H NMR

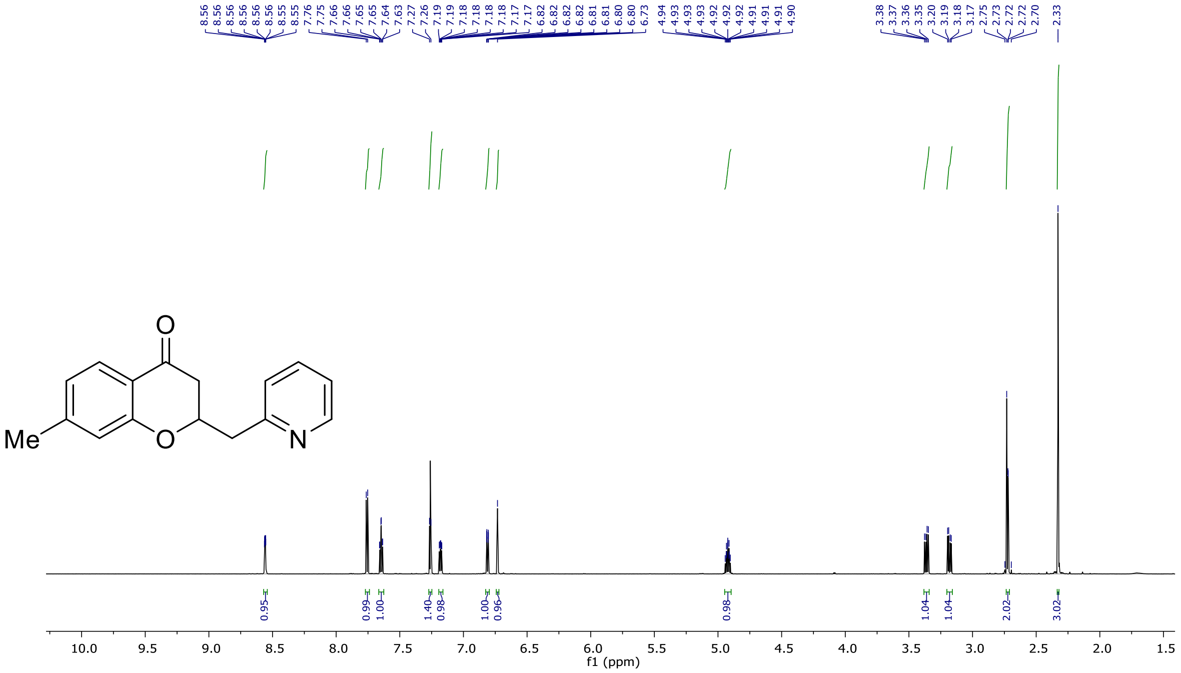

^{13}C NMR

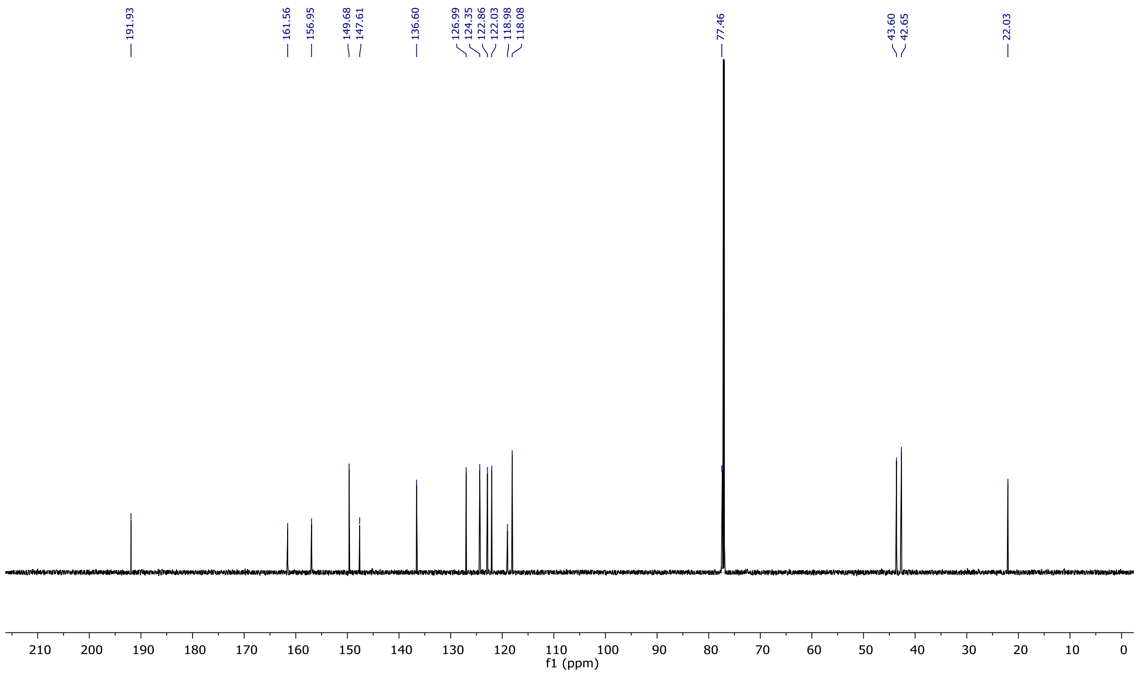

6-Fluoro-2-[(pyridin-2-yl)methyl]-3,4-dihydro-2*H*-1-benzopyran-4-one (5d)

[1] H NMR

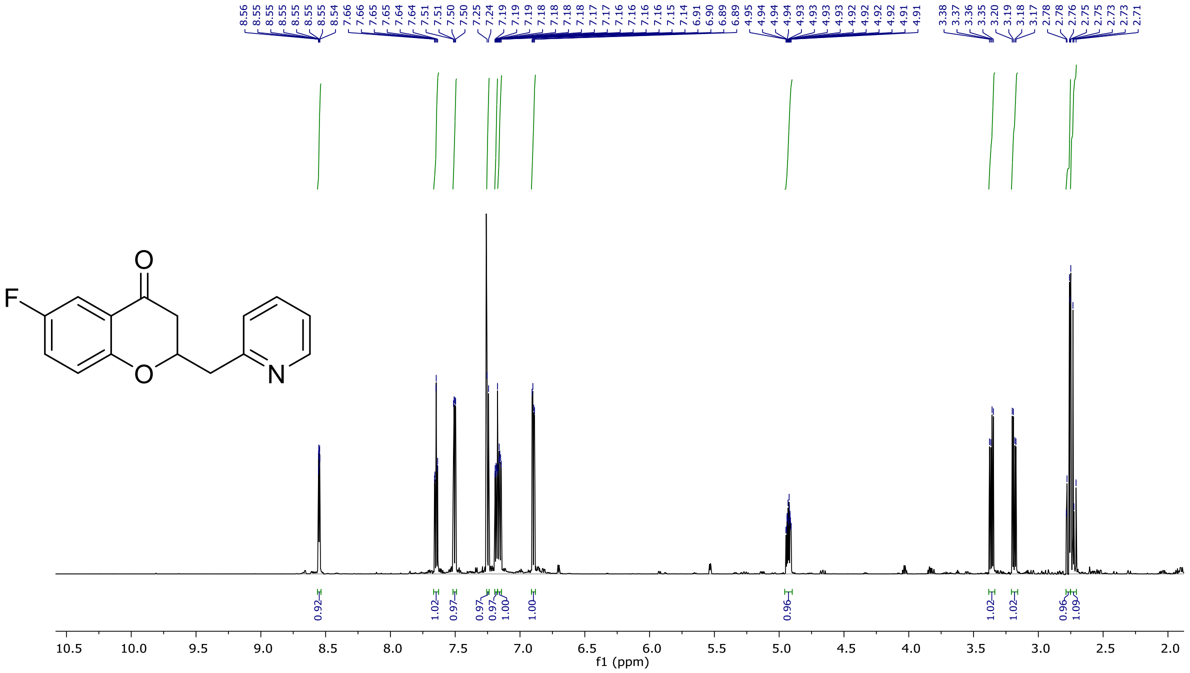

[13] C NMR

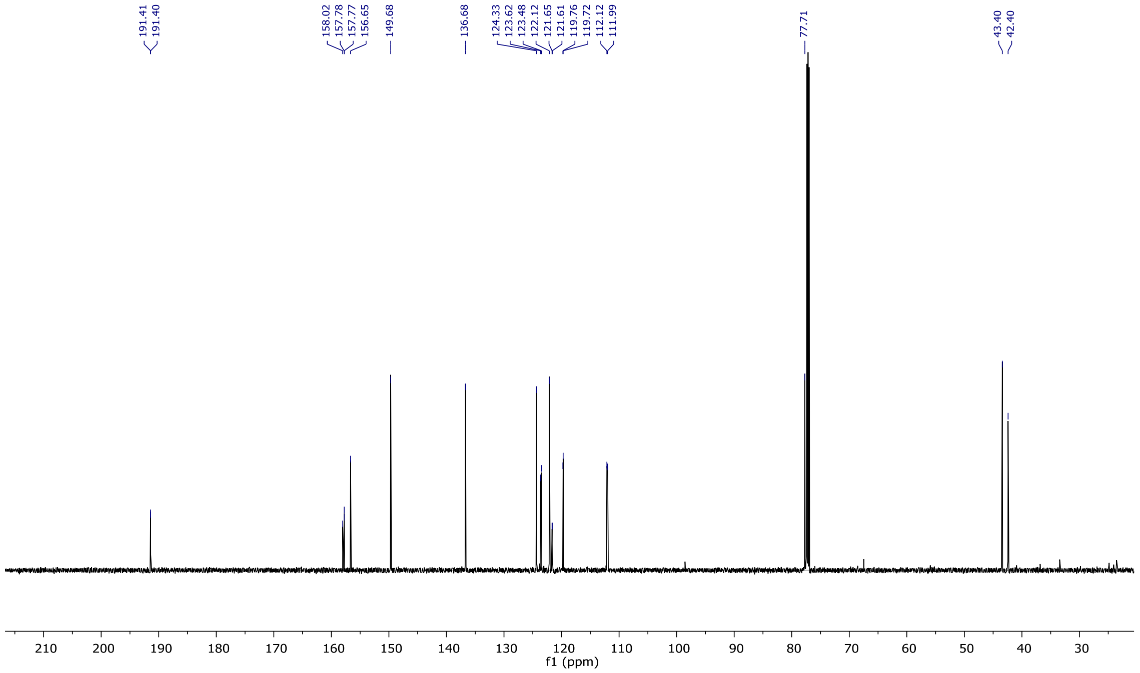

6-Chloro-2-[(pyridin-2-yl)methyl]-3,4-dihydro-2*H*-1-benzopyran-4-one (5e)

^{1}H NMR

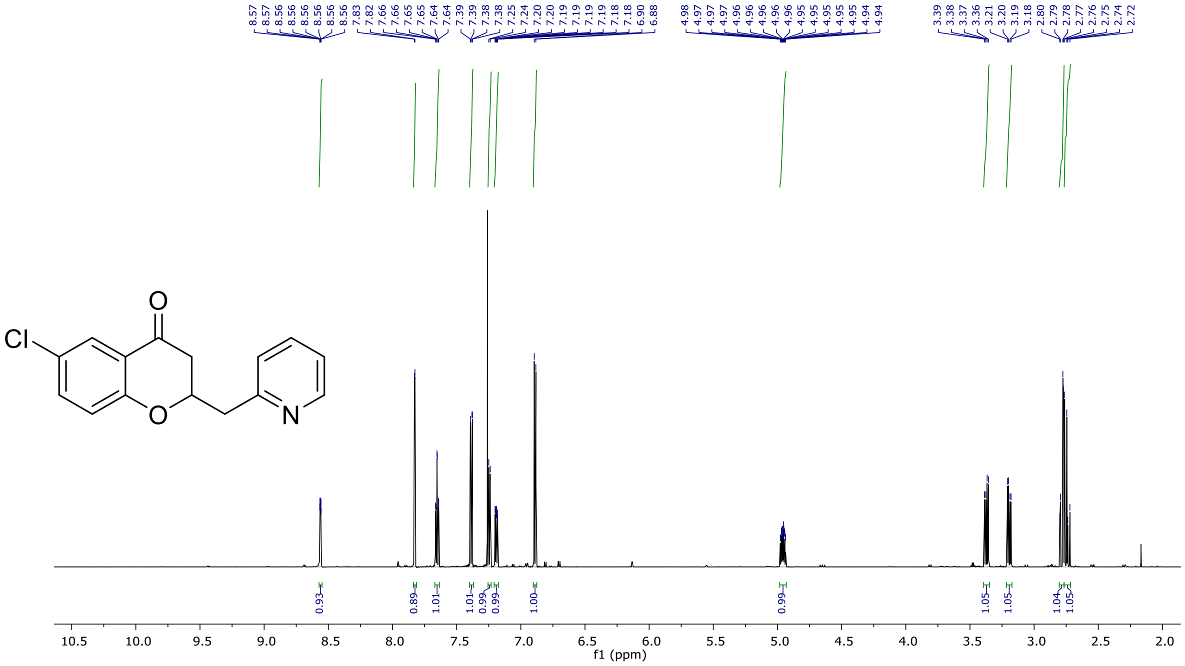

^{13}C NMR

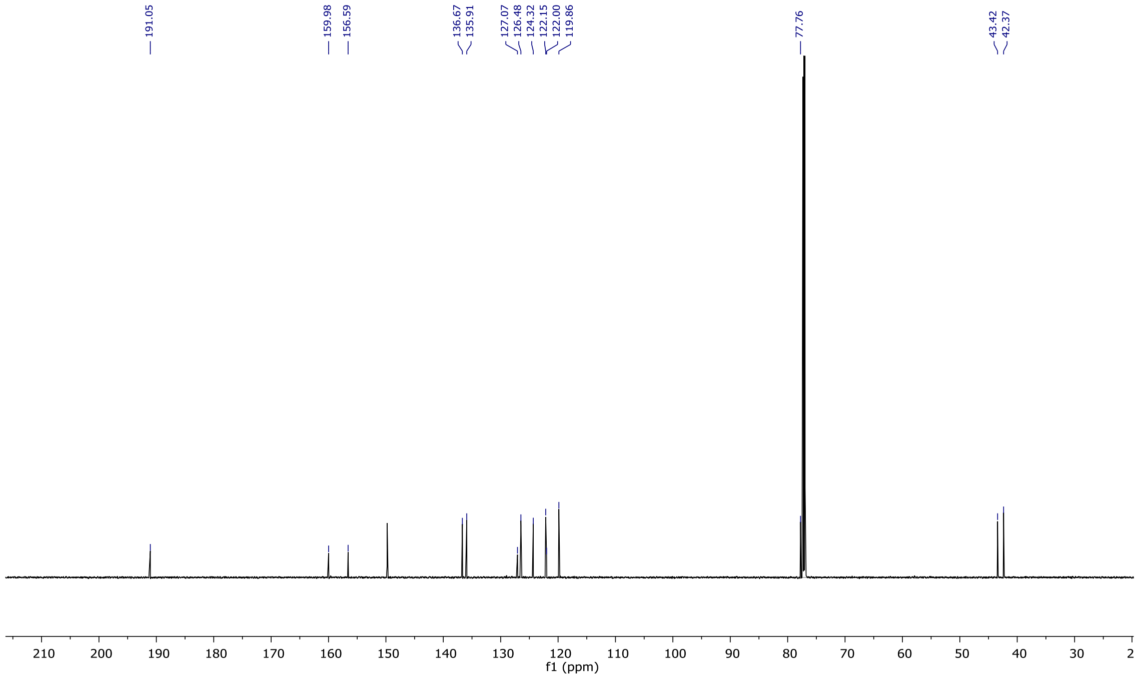

4-[(Pyridin-4-yl)methyl]-3,4-dihydro-2*H*-1-benzopyran-2-one (7)

[1] H NMR

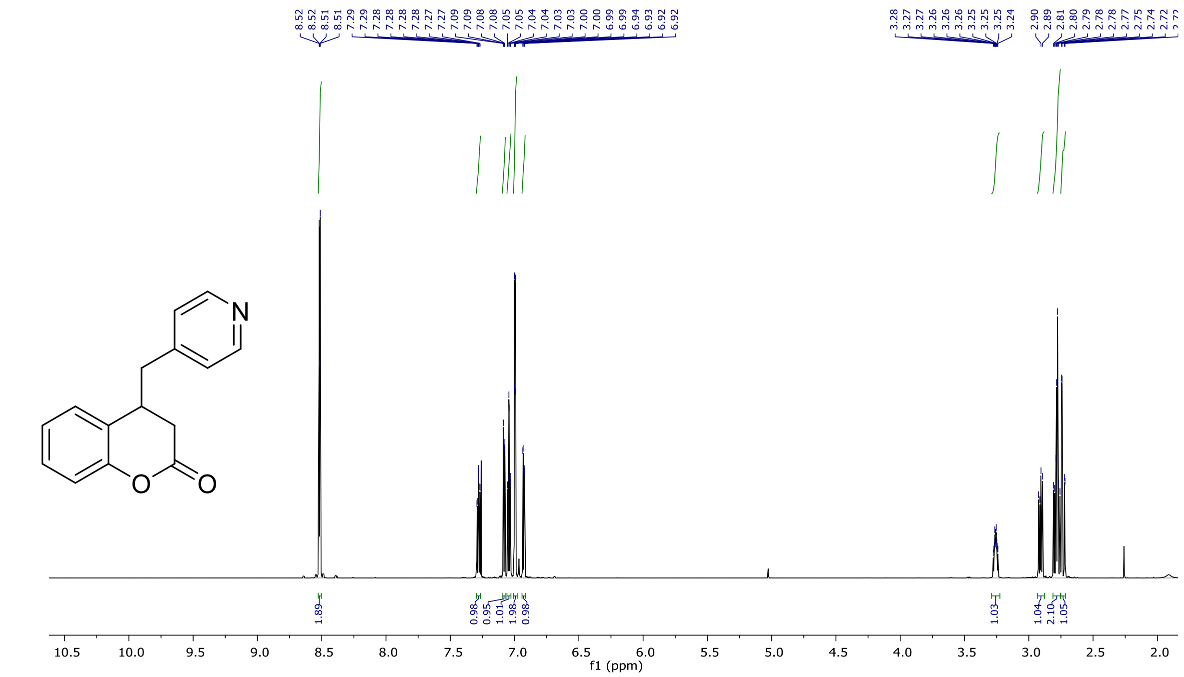

[13] C NMR

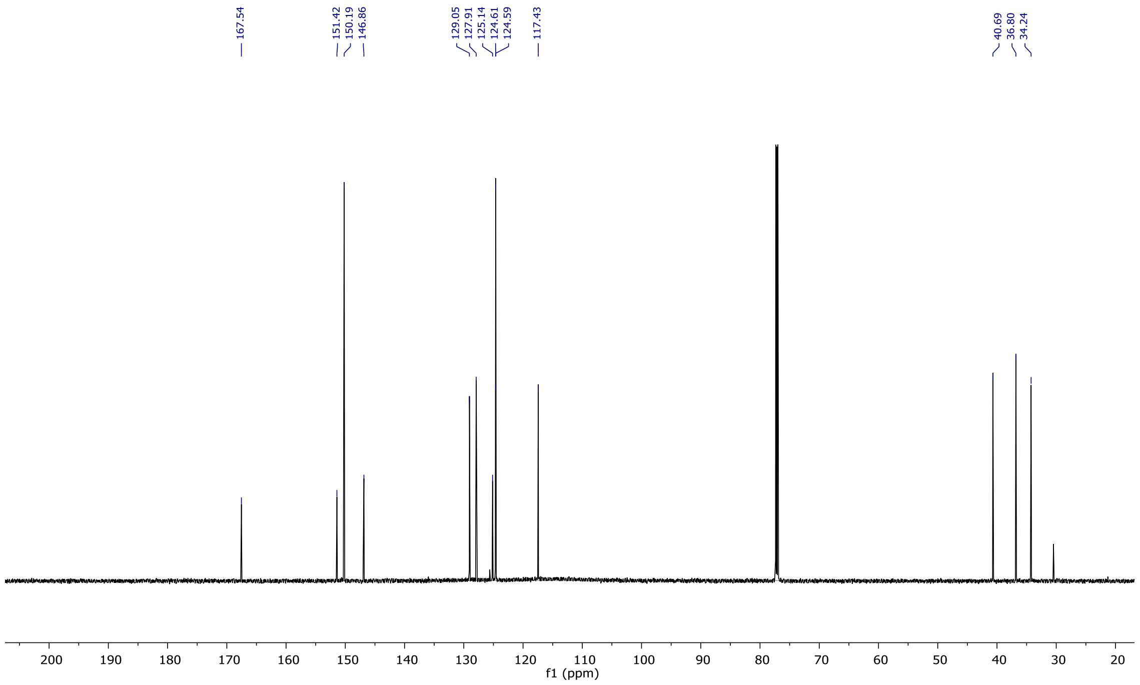

2-[(Pyridin-4-yl)methyl]-3,4-dihydro-2*H*-1-benzopyran-4-one (8)

^{1}H NMR

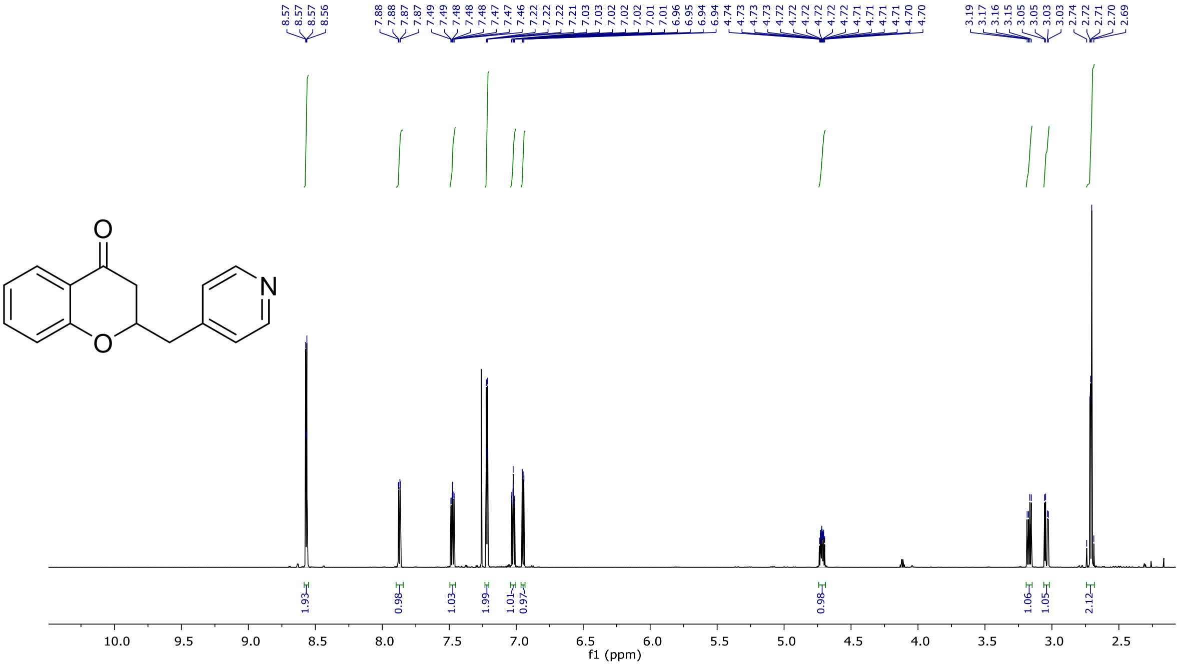

^{13}C NMR

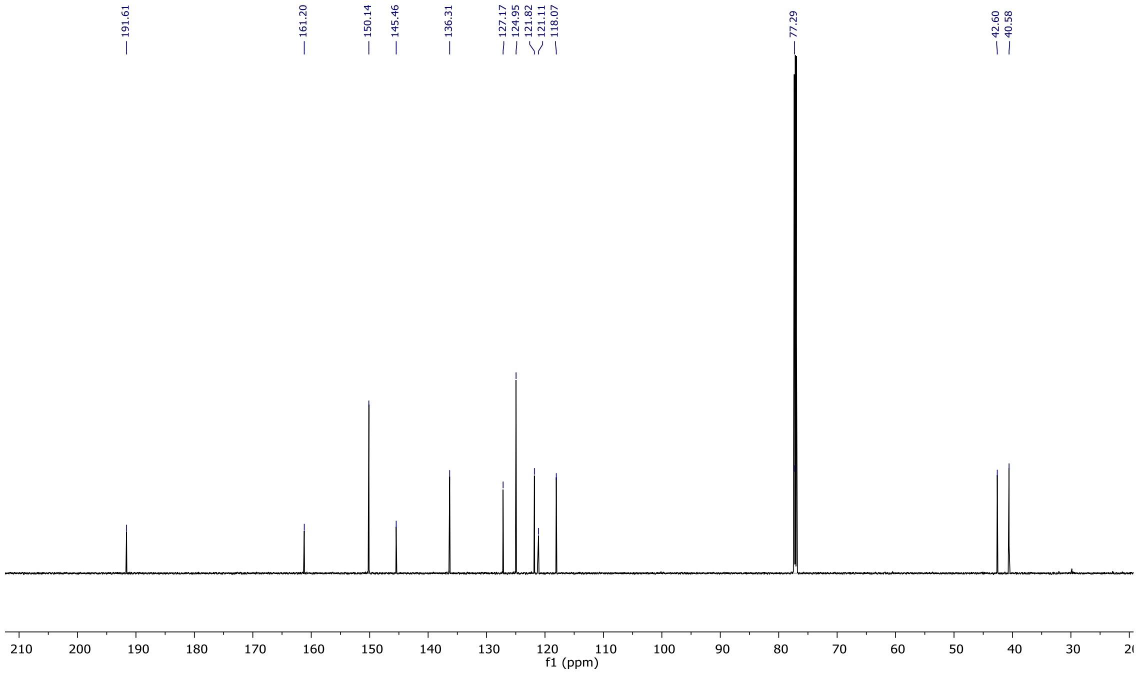

Supplement: Supplementary file 1 [file molecules-26-04689-s001.zip › molecules-1308496-supplementary.pdf]
